# The mitochondrial and chloroplast genomes of the haptophyte *Chrysochromulina tobin* contain unique repeat structures and gene profiles

**DOI:** 10.1186/1471-2164-15-604

**Published:** 2014-07-17

**Authors:** Blake T Hovde, Shawn R Starkenburg, Heather M Hunsperger, Laina D Mercer, Chloe R Deodato, Ramesh K Jha, Olga Chertkov, Raymond J Monnat, Rose Ann Cattolico

**Affiliations:** Department of Genome Sciences, University of Washington, Seattle, WA USA; Los Alamos National Laboratory, Bioscience Division, Los Alamos, NM USA; Department of Biology, University of Washington, Seattle, WA USA; Department of Statistics, University of Washington, Seattle, WA USA; Department of Pathology, University of Washington, Seattle, WA USA

**Keywords:** Haptophytes, Chloroplast genome, Mitochondrial genome, Repeat structure, Repeat function, *Chrysochromulina*

## Abstract

**Background:**

Haptophytes are widely and abundantly distributed in both marine and freshwater ecosystems. Few genomic analyses of representatives within this taxon have been reported, despite their early evolutionary origins and their prominent role in global carbon fixation.

**Results:**

The complete mitochondrial and chloroplast genome sequences of the haptophyte *Chrysochromulina tobin* (Prymnesiales) provide insight into the architecture and gene content of haptophyte organellar genomes. The mitochondrial genome (~34 kb) encodes 21 protein coding genes and contains a complex, 9 kb tandem repeat region. Similar to other haptophytes and rhodophytes, but not cryptophytes or stramenopiles, the mitochondrial genome has lost the *nad*7, *nad*9 and *nad*11 genes. The ~105 kb chloroplast genome encodes 112 protein coding genes, including *ycf*39 which has strong structural homology to NADP-binding nitrate transcriptional regulators; a divergent ‘CheY-like’ two-component response regulator (*ycf*55) and Tic/Toc (*ycf*60 and *ycf*80) membrane transporters. Notably, a zinc finger domain has been identified in the *rpl*36 ribosomal protein gene of all chloroplasts sequenced to date with the exception of haptophytes and cryptophytes - algae that have gained (via lateral gene transfer) an alternative *rpl*36 lacking the zinc finger motif. The two *C. tobin* chloroplast ribosomal RNA operon spacer regions differ in tRNA content. Additionally, each ribosomal operon contains multiple single nucleotide polymorphisms (SNPs) - a pattern observed in rhodophytes and cryptophytes, but few stramenopiles. Analysis of small (<200 bp) chloroplast encoded tandem and inverted repeats in *C. tobin* and 78 other algal chloroplast genomes show that repeat type, size and location are correlated with gene identity and taxonomic clade.

**Conclusion:**

The *Chrysochromulina tobin* organellar genomes provide new insight into organellar function and evolution. These are the first organellar genomes to be determined for the prymnesiales, a taxon that is present in both oceanic and freshwater systems and represents major primary photosynthetic producers and contributors to global ecosystem stability.

**Electronic supplementary material:**

The online version of this article (doi:10.1186/1471-2164-15-604) contains supplementary material, which is available to authorized users.

## Background

Globally, primary producers fix ~100 gigatons of carbon each year [1]. This production is equally distributed between terrestrial and aquatic ecosystems [1]. Haptophytes are globally abundant and important photosynthetic microalgae found in both marine and freshwater environments. Recent estimates indicate that haptophytes alone may represent “…30-50% of total photosynthetic standing stock in the world’s oceans” [2], where they play a key role in carbon fixation. Some haptophyte species are photosynthetic as well as mixotrophic, thus can live in dysphotic zones where light levels are too low to support photosynthesis [3]. This metabolic versatility may contribute to fitness, and help explain haptophyte prevalence within global algal populations.

Haptophyte evolutionary history remains enigmatic. Based on fossil records and 18S rDNA phylogenetic analyses [4, 5], it is estimated that these algae are an ancient lineage, arising over 1.2 billion years ago. Phylogenomic analyses of the plastids of *h*aptophytes, *s*tramenopiles, dinoflagellates (*a*lveolates) and *c*ryptophytes show that the plastids of these four groups, collectively termed “CASH” [6], form a monophyletic grouping descendent from red algal plastids. However, the relationships among CASH plastids remains controversial, as differing topologies are recovered in phylogenetic analyses of chloroplast genes using various methods and loci [7–14] (see Green [15] for review). Aside from the plastid lineage controversy, the haptophyte host lineage may be affiliated with the stramenopile-alveolate-rhizaria (SAR) group [11].

Despite their important ecological roles and interesting evolutionary history, there has been little genomic characterization of diverse haptophyte species. Two classes define Haptophytes. The monophyletic Pavlovophyceae display minimal diversity, being described by 4 orders. In contrast, the polyphyletic and globally abundant Prymnesiophyceae encompass 6 orders, of which the B2 clade seems most dominant in marine and fresh water ecosystems [16]. Of this vast assemblage of haptophytes, the organellar genomes of only one representative of the Pavlovophyceae, (*Pavlova lutheri*: Pavlovales), and three of the Prymnesiophyceae (*Emiliania huxleyi*: Isochrysidales; *Phaeocystis antarctica* and *Phaeocystis globosa*: Phaeocystales) have been sequenced. The large and complex Prymnesiales that encompass the B1 to B5 clades [17, 18], lack a sequenced representative. This omission is surprising given reports demonstrating that >55% of all haptophyte sequences in a Mediterranean location belong to this taxonomic assemblage [16, 19], and that members of this clade can dominate fresh water ecosystems [20]. We reasoned that determining the genomic sequence of a B2 representative in the Prymnesiales would provide new information on haptophyte evolutionary origins and ecosystem roles.

The B2 clade prymnesiophyte chosen for sequencing, *Chrysochromulina tobin,* is a newly defined algal species (Deodato, Barlow, Hovde et al. in prep). This small (4 μm) unicellular alga is naturally wall-less, being delineated solely by a plasma membrane. It lacks scales or additional extracellular structures. *Chrysochromulina tobin* lives in fresh to brackish water and is mixotrophic [21], using a long haptonema to hunt bacterial prey. Bacteria-containing cultures exhibit improved growth and produce more fatty acid than those maintained axenically (Deodato, Barlow, Hovde et al. in prep). Nevertheless, *C. tobin* can be grown on completely defined artificial medium, and cell division is synchronized by light/dark photoperiods.

Here we report the sequencing and annotation of the complete *Chrysochromulina tobin* mitochondrial and chloroplast genomes. These genomes were analyzed using available data from rhodophytes (red algae), chlorophytes (green algae) as well as haptophytes and other members of the CASH complex. Data reported here show the mitochondrial genome to contain a large and complex repeat comprising 28% of the mitochondrial sequence, and to have lost several *nad* genes (*nad*7*,* 9 and 11). The *C. tobin* chloroplast genome contains a novel intergenic ribosomal spacer region, and multiple SNPs between rDNA copies within the inverted ribosomal repeat regions. Analyses of chloroplast tandem and inverted repeats demonstrate gene-specific associations, regardless of algal species. Features of several genes provide new insight into aspects of chloroplast genome evolution including lateral gene transfer, gene retention, novel functional rolls and putative regulatory structures localized within intergenic regions.

## Results and discussion

### Mitochondrial and chloroplast genome sequencing

Purified total genomic DNA was used to prepare libraries for both the 454 pyrosequencing and Illumina platforms. A total of 4.7 million reads and 79 million reads were generated on the 454 and Illumina platforms respectively, and then assembled using Newbler [22] and Velvet [23] (see Methods). The resulting draft assembly included 3,472 contigs with an average length of ~17 kb. A single contig of 25,263 bp represented 74% of the mitochondrial genome, but no other assembled contigs contained remaining known mitochondrial sequence, likely due to the presence of a large repeat structure. This repeat structure required PCR amplification and sequencing to resolve the final circular draft. The chloroplast genome was contained in two assembled contigs that totaled 101,192 bp in length. Due to the ribosomal inverted repeat, PCR followed by Sanger sequencing of the amplified products was used to join the two sequences and form a complete, circular mapping chloroplast assembly.

### Mitochondrial gene content

**Figure 1:**
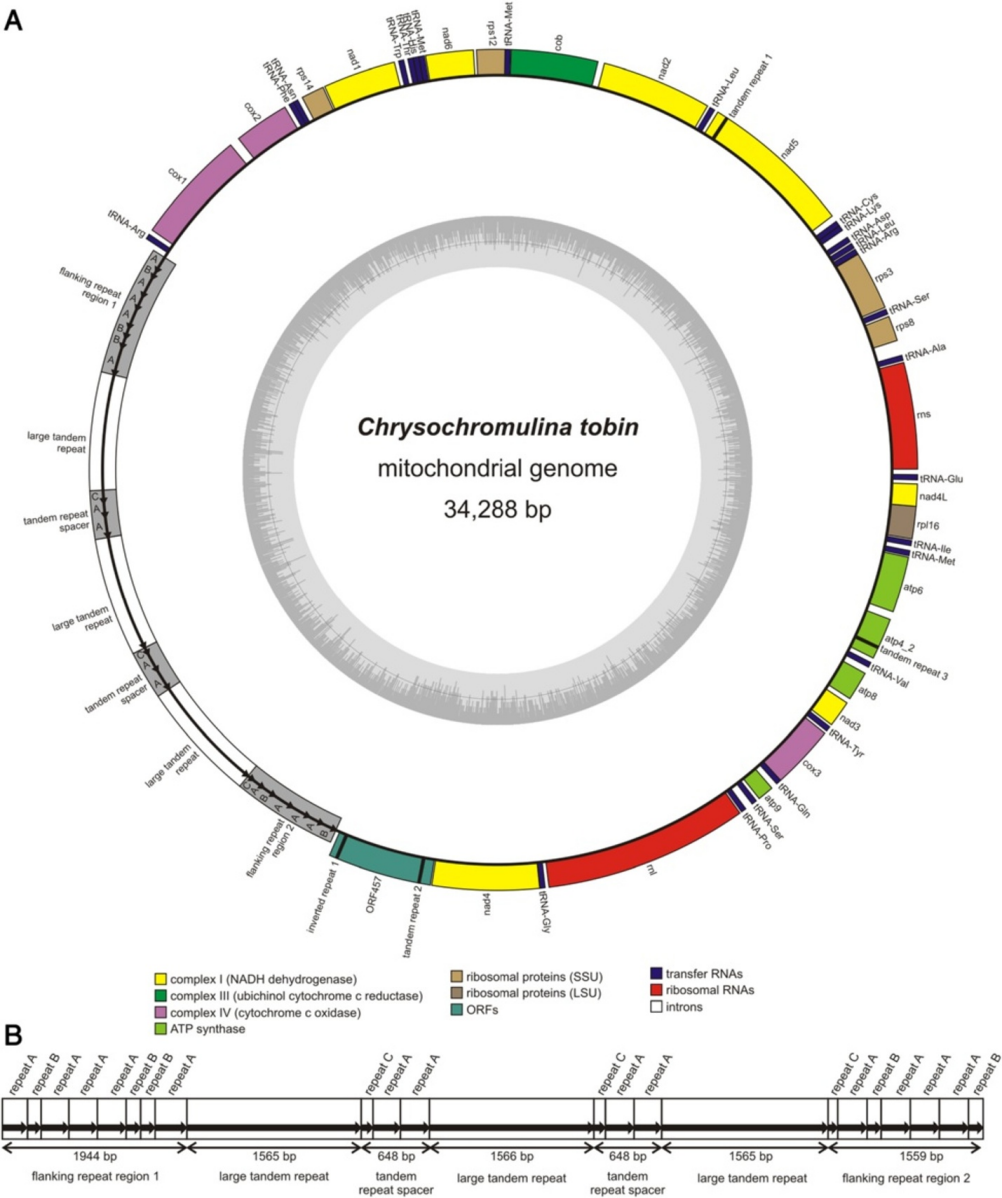
***Chrysochromulina tobin*** **mitochondrial genome map. (A)** All genes are transcribed in the same direction. A split ribosomal operon is present. The large repeat region of 9495 bp represents a significant portion (~28%) of the genome. **(B)** Detailed representation of the complex repeat region found in the mitochondrial genome. Three large tandem repeat regions are flanked by two sections containing small tandem repeats designated A, B and C. These repeat subunits also make up the regions separating the large tandem repeats from each other. Blocks A, B and C have strong, but rarely perfect, sequence identity.

The *Chrysochromulina tobin* mitochondrial genome [GenBank:KJ201908] is 34,288 bp in size, has a GC content of 31.4%. The genome encodes 48 genes, including 25 tRNAs, 21 protein coding genes and a split ribosomal operon comprising the 16S and 23S rRNA genes (Figure 1). The mitochondrial 21 protein coding gene complement includes a single novel open reading frame (orf457) that encodes a 457 amino acid protein that lacks strong homology to any other protein within the NCBI database. As in other sequenced haptophytes, NCBI translation table 4 [24] was used, where UGA codes tryptophan rather than a termination codon. Comparison of the genomic content among all published haptophyte genomes (*E. huxleyi*[25, 26]: [GenBank:AY342361, JN022704]; *P. antarctica*[27]: [GenBank:JN131834, JN131835]; *P. globosa*[27]: [GenBank:KC967226]; *P. lutheri*: [GenBank:HQ908424]) indicate that 14 energy and metabolism genes are conserved in all examined taxa. All haptophyte genomes also contain an identical complement of five ribosomal proteins (*rps*3*, rps*8*, rps*12*, rps*14*,* and *rpl16*) except for *P. antarctica* and *P. globosa* which are missing the *rps*8 or the *rps*8 and *rps*14 genes respectively (Table 1). Most notably, *nad*7, *nad*9 and *nad*11 are consistently missing from all haptophyte and rhodophyte mitochondrial genomes sequenced to date. Interestingly, these three genes are present in all cryptophyte and stramenopile mitochondrial genomes.

**Table 1:** **Comparison of haptophyte mitochondrial genomes**

	***Chrysochromulina tobin*** UWC 291	***Phaeocystis antarctica*** (Partial) CCMP 1374	***Phaeocystis globosa*** (Partial) Pg-G(A)	***Emiliania huxleyi*** CCMP 1516	***Emiliania huxleyi*** CCMP 373	***Pavlova lutheri*** CCMP 1325 (Partial)
Genome Size (bp)	34288	27547	24477	28660	29013	34086
GC %	31.4	29.7	30.5	28.5	28.3	37.3
**Protein-coding genes**	21	19	18	20	20	22
Respiratory coding proteins	15	15	15	14	14	15
Ribosomal proteins	5	4	3	5	5	5
Unique gene content	ORF457	-	-	*dam*	*dam*	ORF636(*dam*), ORF105
Missing genes found in other haptophytes	-	*rps8*	*rps8*, *rps14*	*atp8* (partial only)	*atp8* (partial only)	-
**RNA-coding genes**						
tRNAs	25	26	25	25	25	24
rRNA content	1 (split operon)	1 (split operon)	1 (split operon)	1 (intact operon)	1 (intact operon)	1 (split operon)
**Repeat elements**						
Tandem repeats	3	27	4	5	7	27
Inverted repeats	1	4	6	3	1	1
Large repeat regions	1	2	2	1	1	1

Note: No introns were found within any of the listed genomes.

With respect to mitochondrial gene synteny among haptophyte taxa, many structural rearrangements have occurred. The results of Mauve [28] gene cluster analysis showed very poor gene cluster conservation (Additional file 1). The extensive nature of shuffled gene order is further evidenced by the fact that the ribosomal operon is split in all haptophyte genomes (*C. tobin*, *P. antarctica, P. globosa, and P. lutheri*) except *E. huxleyi*.

### Mitochondrial repeats

The *Chrysochromulina tobin* mitochondrial genome contains a large repeat region measuring 9.3 kb in length (Figure 1B). This region features three large tandem repeats, each ~1.5 kb in length that are flanked by two regions consisting of additional small tandem repeats. These small tandem repeat regions are composed of three subunits, arbitrarily classified A, B and C, based on sequence homology (though all sequences within each subunit class are not 100% identical). Repeat unit A is comprised of 290 bp. Unit B consists of 156 bp, of which ~84 bp exhibit significant sequence identity to unit A. Unit C is 85 bp in length. Although the flanking repeat regions are not identical in size (regions 1 and 2 are 1896 bp and 1558 bp respectively), a consistent pattern of B-A-A-A-B is found within these two flanking domains. The three large tandem repeats are separated from each other within the repeat region by spacers (consisting of a C-A-A pattern). Interestingly, this direct repeat arrangement is strikingly similar to the larger (35 kb) repeat structure found in the diatom *Phaeodactylum tricornutum*[29]. The cryptophytes, *Hemiselmis andersenii* and *Rhodomonas salina*, and the chlorophytes *Pedinomonas minor* and *Acutodesmus obliquus* also contain large tandem repeat regions (>4 kb) that differ from the minimal repeat embellishment seen in most mitochondrial genomes of other algae.

Not surprisingly, the complexity of this repeat structure caused assembly challenges*.* The fact that *P. antarctica, P. globosa,* and *P. lutheri* mitochondrial genomes remain incomplete is likely due to the presence of one or more large repeat structures. For example, Smith et. al. [27] reported unresolved repeats within two repeat regions in *P. antarctica* and *P. globosa*. Unfortunately, the use of short read, high throughput sequencing techniques do not easily facilitate solving these complex repeat structures. The first *E. huxleyi* mitochondrial genome published in 2004 [25], the stramenopile, *Heterosigma akashiwo*[30], as well as the diatoms *Phaeodactylum tricornutum* and *Thalassiosira pseudonana*[29] utilized fosmid sequencing, that supported assembly and primer walking for the resolution of longer repeats.

### Chloroplast gene content

**Figure 2:**
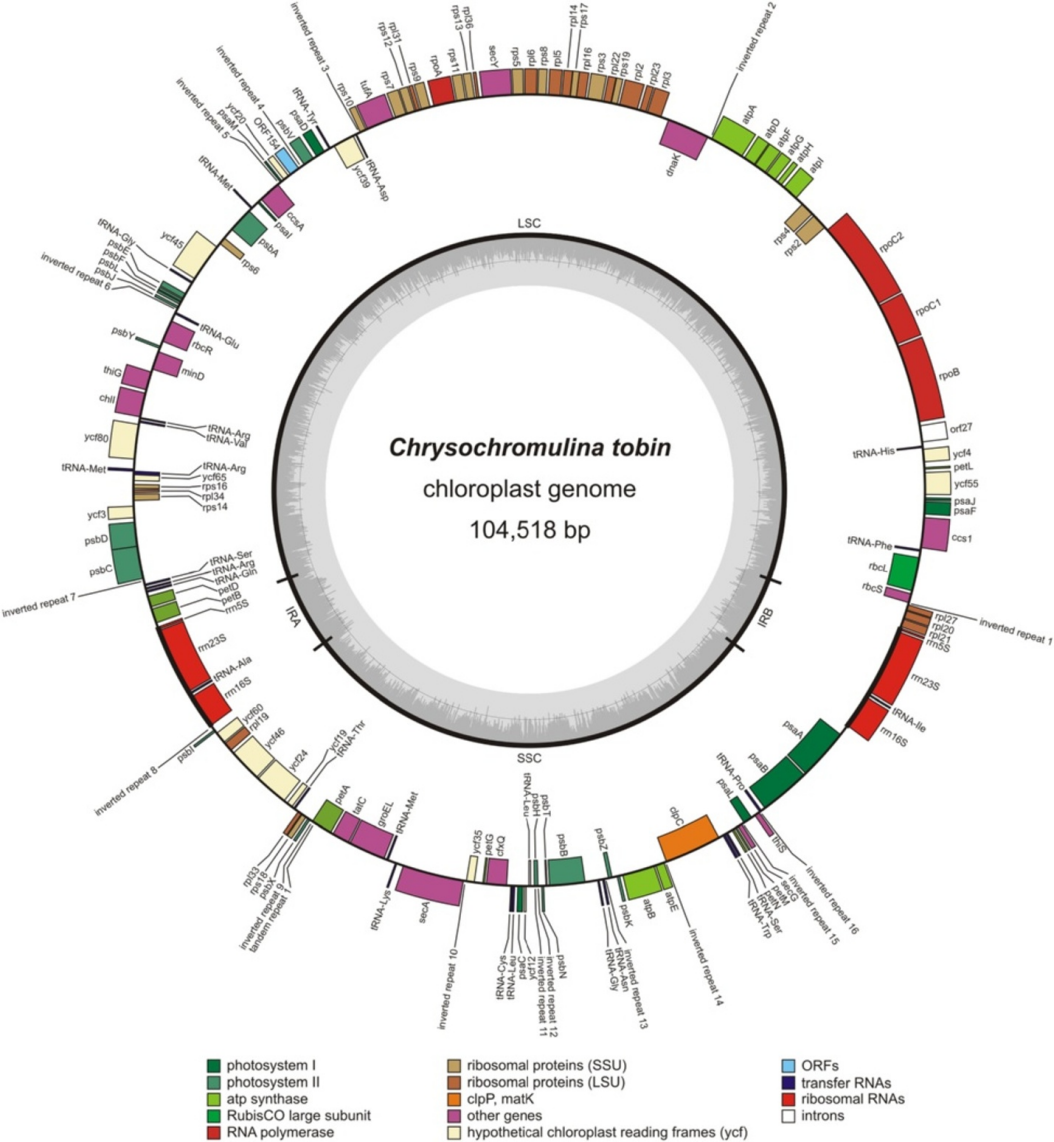
***Chrysochromulina tobin*** **chloroplast genome map.** Genes facing outside are transcribed in the counter-clockwise direction and genes facing inside are transcribed in a clockwise direction. Two copies of the ribosomal operon are inverted and the repeat region contains no other genes beyond the ribosomal subunits. The small single copy (SSC) and large single copy (LSC) regions are labeled. Inverted and tandem repeats are also designated.

The *Chrysochromulina tobin* chloroplast genome [GenBank:KJ201907] is 104,518 bp in size and has a GC content of 36.3%. The genome encodes 145 genes (Figure 2) including 27 tRNA coding genes, 112 protein coding genes and an inverted repeat, with each repeat copy containing the 23S, 16S and 5S rRNA genes. The tRNAs present in this genome represent all potential amino acid anticodons, including a start methionine. Within the chloroplast genome, codon usage is standard for plastid genomes, and protein alignments suggest that codon GTG (valine) may serve as the translation initiation codon for *ycf*55, *rps*3, *psb*E, *ycf*65 and *psb*C. Such alternative translational start codons have been reported to occur in algal chloroplast genomes of wide taxonomic divergence (e.g*., Cyanidium caldarium, Odontella sinensis, Heterosigma akashiwo,* and *Emiliania huxleyi*) [31–33] although not for the same genes established in *C. tobin*.

The *C. tobin* chloroplast gene complement is similar to other sequenced haptophyte chloroplast genomes: (Table 2; *E. huxleyi*[26, 31]: [Genbank:AY741371, JN022705]; *P. antarctica*: [GenBank:NC_016703]; *P. globosa*[27]: [GenBank:NC_021637] and *P. lutheri*: [GenBank: NC_020371] [6]). Additionally, an “uncultured prymnesiophyte C19847” (derived from metagenomic data of oceanic samples collected from the North Atlantic [GenBank:HM565909] [34]) was included in this analysis. All haptophyte genomes are relatively small in size when compared to other microalgal species (Additional file 2). Gene content comparison shows *E. huxleyi* (113 protein-coding genes) contains *dfr* (a two component signaling protein) that is absent in *C. tobin.* Unlike *C. tobin, P. antarctica* and *P. globosa* chloroplast genomes (both having 108 genes) are missing ORF132 (unknown function), *ycf20* (unknown function), *thi*G, and *thi*S (thiamine biosynthesis protein G and S respectively). A conserved coding region (ORF154), found uniquely in *C. tobin* (154 amino acids) and *E. huxleyi* (132 amino acids), is located adjacent to *psb*V in both genomes. The amino acid identity of these two hypothetical genes is low, suggesting remnants of functional proteins. The chloroplast genome of the phylogenetically distant haptophyte, *P. lutheri*, is missing 7 genes, and contains an additional 9 genes that are not found in available haptophyte chloroplast genomes (Table 2). While gene content is similar among all of the haptophytes analyzed, the freshwater *C. tobin* actually has the highest sequence identity to the marine uncultured prymnesiophyte C19847. This result is not too surprising given recent studies that document the occurrence of multiple, independent freshwater colonizations by haptophytes [20, 35]. Our 18 s rDNA based phylogenetic analyses (Deodato, Barlow et al., in prep) show *C. tobin* to cluster with species isolated from fresh water lakes in France [20].

**Table 2:** **Comparison of haptophyte chloroplast genomes**

	***Chrysochromulina tobin*** UWC 291	***Phaeocystis antarctica*** CCMP 1374	***Phaeocystis globosa*** Pg-G(A)	***Emiliania huxleyi*** CCMP373	***Emiliania huxleyi*** CCMP1516	***Pavlova lutheri*** CCMP 1325	Uncultured Prymnesiophyte C19847 (Partial genome)
Genome Size (bp)	104518	105651	107461	105309	105297	95281	45567
GC %	36.3	35.5	35.4	36.8	36.8	35.6	37.2
**Protein-coding Genes**	112	108	108	119	113	111	45
Unique gene content	ORF154, *ycf20*, *thiG*, *thiS*			ORF132, *ycf20*, *dfr*, *thiG*, *thiS*		ORF140, *ycf66*,ORF208, ORF66, rpoZ, ORF84, RF489,ORF70, ORF69	N/A
Missing genes found in other haptophytes						*ycf55, ycf47, ycf80, ycf65, rpl34,ycf46, ycf35*	N/A
**RNA-coding genes**							
tRNAs	27	27	27	28	30	27	18
Ribosomal operons (23S, 16S, 5S)	2 (inverted repeat)	2 (inverted repeat)	2 (inverted repeat)	2 (inverted repeat)	2 (inverted repeat)	1	1
**Repeat elements**							
Inverted repeats	16	7	10	15	16	6	11
Tandem repeats	1	6	2	1	1	2	1

Note: No introns were found within any of the listed genomes.

**Figure 3:**
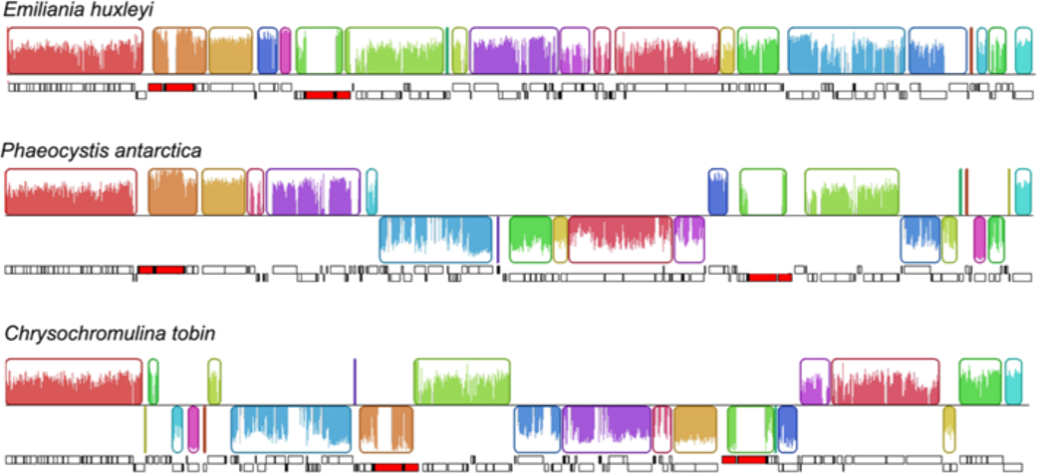
**Gene map comparison of** ***C. tobin, P. antarctica*** **and** ***E. huxleyi*** **chloroplast genomes aligned using Mauve.** Inside each block a sequence identity similarity profile is shown. Individual genes and strandedness are shown below each genome block. Red regions in the individual gene plots indicate the locations of the large ribosomal operon repeat regions.

Co-linearity in gene placement among haptophyte chloroplast genomes was assessed (Figure 3). Unlike in diatoms [36], gene clusters have been exchanged between the large and small single copy regions within these haptophyte chloroplast genomes. When comparing *E. huxleyi* to *C. tobin*, and *E. huxleyi* to *P. antarctica*, 17 and 13 gene clusters were conserved, respectively. A highly conserved region of 20,610 bp encompassing *ccs*1 through *atp*A (18 genes) was identified. This region contains a single inversion in the *C. tobin rps*2 and *rps*4 coding region, and is more highly conserved between *E. huxleyi* and *P. antarctica* – expanding to a ~30,000 bp region that initiates with *pet*L (cytochrome b6/f complex component) and ends with *psb*E (photosystem II protein). Another large gene cluster conserved in all three species consists of ~15,000 bp that contains the commonly preserved 24 ribosomal protein gene operon and the *dna*K heat shock protein. GRIMM [37] analysis was used to quantify the degree of gene rearrangement among the three completed haptophyte chloroplast genomes above. The most parsimonious result found 11 genome rearrangements occurring between *E. huxleyi* and *P. antarctica*, 10 rearrangements between *P. antarctica* and *C. tobin*, and 19 rearrangements between *E. huxleyi* and *C. tobin.* The scrambled placement of genes among these haptophytes yields no clear insight into relatedness.

### Chloroplast repeats

#### Large inverted repeat

**Figure 4:**
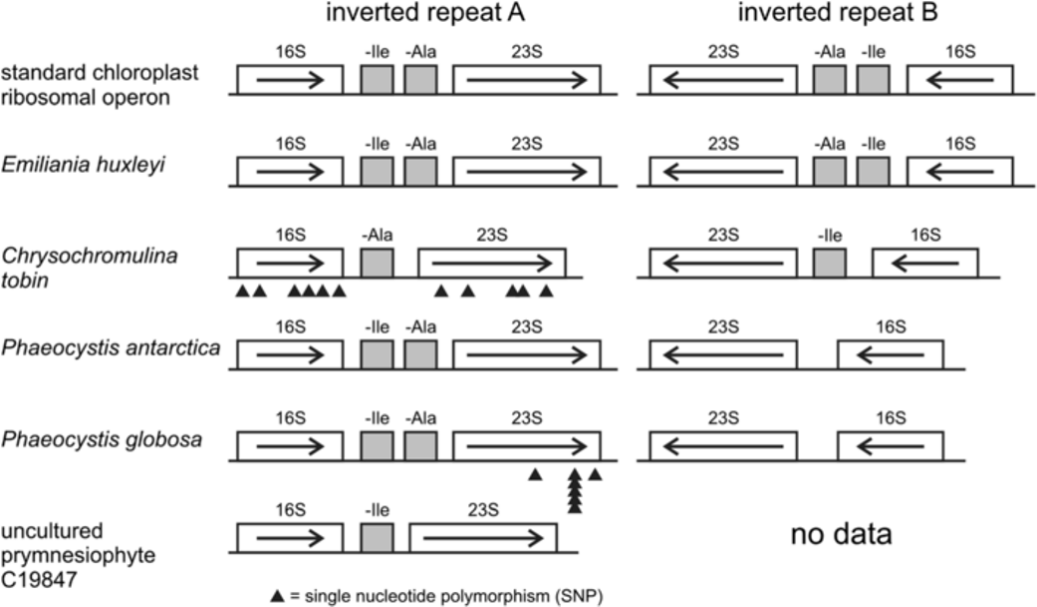
**Ribosomal operon repeats of** ***C. tobin*** **and other haptophyte chloroplast genomes.** The “standard” chloroplast ribosomal operon contains tRNA-Ile and tRNA-Ala in the ribosomal intergenic spacer regions (ISR). *Chrysochromulina tobin* contains tRNA-Ala in operon A and tRNA-Ile in operon B. Multiple SNPs (black triangles) are present between the *C. tobin* large and small ribosomal subunits coding regions to each other. One copy of the ribosomal operon contains both tRNAs while the other operon lacks both tRNAs in *P. antarctica* and *P. globosa* plastid genomes. A metagenomic sample, uncultured prymnesiophyte C19847 [28], contains a single tRNA-Ala; the second ribosomal copy was not assembled and is therefore unknown in structure.

The *Chrysochromulina tobin* ribosomal repeat region is structurally unique when compared to those found in both land plants and all algal chloroplasts sequenced to date. Eighty two percent (209/256) of all chloroplast genomes (including non-algal species) examined at the genus level contain a large inverted repeat. The conventional structure of this conserved operon includes the 16S ribosomal gene, an intergenic spacer region (ISR) that encodes the tRNA-isoleucine (anticodon GAU), and the tRNA-alanine (anticodon UGC). The ISR is followed by the 23S ribosomal subunit gene and the 5S ribosomal gene (Figure 4). In land plants and chlorophytic algae, and less often in rhodophytes and CASH members [33, 38], the repeat region expands to include additional genes that flank the ribosomal gene operon, making the inverted repeat in chlorophytes larger on average (Table 3). The ribosomal inverted repeat structures in *C. tobin* have non-identical tRNA coding sequences within each ribosomal intergenic spacer region (detail shown in Additional file 3). This domain normally contains two identical tRNA coding regions in each inverted repeat. However, *C. tobin* has only tRNA alanine in inverted repeat A, and tRNA isoleucine in inverted repeat B (Figure 4). This pattern is also likely present in the uncultured prymnesiophyte C19847. Only a single operon was assembled for this organism, but that operon solely contained tRNA-Ile within the ISR. Interestingly, the C19847 ribosomal operon that is adjacent to the *pet*B gene contains a tRNA-Ile, while the ribosomal operon adjacent to *pet*B in *C. tobin* contains tRNA-Ala. Intramolecular recombination within chloroplast genomes having an inverted repeat is well documented for several algae as well as in land plants [39–41]. This process generates genomic isomers that differ solely in the orientation of their single copy domains. Surprisingly, using long PCR to bridge both *C. tobin* repeat regions, no evidence of such flip-flop recombination was found.

**Table 3:** **Inventory of algal inverted repeat sequences in chloroplast genomes**

Organism	SNPs or indels found in 23S ribosomal subunit	SNPs or indels found in 16S ribosomal subunit	Inverted repeat length ^†^	Accession number
**Haptophytes**				
*Chrysochromulina tobin*	5	6	4656	This work
*Emiliania huxleyi* CCMP373	0	0	4674	NC_007288
*Emiliania huxleyi* CCMP1516	0	0	4868	JN022705
*Phaeocystis antarctica*	0	0	4674	JN117275
*Phaeocystis globosa*	8	0	4611	NC_021637
*Pavlova lutheri*	No Inverted Repeat			NC_020371
**Stramenopiles**				
*Apedinella radians*	2	2	4732	Unpublished*
*Aureococcus anophagefferens*	No Inverted Repeat			NC_012898
*Aureoumbra lagunensis*	No Inverted Repeat			NC_012903
*Botrydium cystosum*	0	0	4924	Unpublished*
*Ectocarpus siliculosus*	0	0	8616	NC_013498
*Fistulifera sp.* JPCC DA0580	0	0	12031	NC_015403
*Fucus vesiculosus*	0	0	5242	NC_016735
*Heterosigma akashiwo*	0	0	21665	NC_010772
*Nannochloropsis gaditana*	0	0	5109	NC_020014
*Nannochloropsis oculata*	4	5	7541	Unpublished*
*Nannochloropsis salina*	0	0	5131	Unpublished*
*Nereocystis luetkeana*	0	0	5416	Unpublished*
*Odontella sinensis*	0	0	7725	NC_001713
*Pelagomonas calceolata*	No Inverted Repeat			Unpublished*
*Phaeodactylum tricornutum*	0	0	6916	NC_008588
*Pinguiococcus pyrenoidosus*	0	0	5070	Unpublished*
*Saccharina japonica*	5	1	5312	NC_018523
*Synedra acus*	1	0	6795	NC_016731
*Synura petersenii*	0	0	22465	Unpublished*
*Thalassiosira oceanica* CCMP1005	0	0	23698	NC_014808
*Thalassiosira pseudonana*	0	0	18345	NC_008589
*Tribonema aequale*	0	3	5749	Unpublished*
*Vaucheria litorea*	0	0	4935	NC_011600
***Cryptophytes***				
*Cryptomonas paramecium*	No Inverted Repeat			NC_013703
*Guillardia theta*	3	2	4922	NC_000926
*Rhodomonas salina*	6	2	4959	NC_009573
***Chlorophytes***				
*Acutodesmus obliquus*	0	0	12023	NC_008101
*Bryopsis hypnoides*	No Inverted Repeat			NC_013359
*Chlamydomonas renhardii*	0	0	22211	NC_005353
*Chlorella variabilis*	No Inverted Repeat			NC_015359
*Chlorella vulgaris*	No Inverted Repeat			NC_001865
*Coccomyxa subellipsoidea* C-169	No Inverted Repeat			NC_015084
*Dunaliella salina*	0	0	14409	NC_016732
*Floydiella terrestris*	No Inverted Repeat			NC_014346
*Gonium pectorale*	0	0	14750	NC_020438
*Leptosira terrestris*	No Inverted Repeat			NC_009681
*Micromonas sp.* RCC299	0	0	7307	NC_012575
*Monomastix sp.* OKE-1	No Inverted Repeat			NC_012101
*Nephroselmis olivacea*	0	0	46137	NC_000927
*Oedogonium cardiacum*	0	0	35492	NC_011031
Oltmannsiellopsis viridis	0	0	18510	NC_008099
*Ostreococcus tauri*	1	0	6825	NC_008289
*Parachlorella kessleri*	0	0	10913	NC_012978
*Pedinomonas minor*	0	0	10639	NC_016733
*Picochlorum sp.*	No Inverted Repeat			Unpublished*
*Pleodorina starrii*	0	0	16608	NC_021109
*Pseudendoclonium akinetum*	0	0	6110	NC_008114
*Pycnococcus provasolii*	No Inverted Repeat			NC_012097
*Pyramimonas parkeae*	0	0	12865	NC_012099
*Schizomeris leibleinii*	No Inverted Repeat			NC_015645
*Stigeoclonium helveticum*	No Inverted Repeat			NC_008372
*Trebouxiophyceae sp.* MX-AZ01	No Inverted Repeat			NC_018569
**Rhodophytes**				
*Calliarthron tuberculosum*	No Inverted Repeat			NC_021075
*Chondrus crispus*	No Inverted Repeat			NC_020795
*Cyanidioschyzon merolae* strain 10D	No Inverted Repeat			NC_004799
*Cyanidium caldarium*	No Inverted Repeat			NC_001840
*Gracilaria tenuistipitata var. liui*	No Inverted Repeat			NC_006137
*Grateloupia taiwanensis*	No Inverted Repeat			NC_021618
*Porphyra purpurea*	11	20	4827	NC_000925
*Pyropia haitanensis*	1	7	4828	NC_021189
*Pyropia yezoensis*	9	6	4827	NC_007932
**Euglenoids**				
*Euglena gracilis*	0	0	6127	NC_001603
*Euglena viridis*	No Inverted Repeat			NC_020460
*Eutreptiella gymnastica*	No Inverted Repeat			NC_017754
*Monomorphina aenigmatica*	No Inverted Repeat			NC_020018
**Streptophytes**				
*Chaetosphaeridium globosum*	0	0	12431	NC_004115
*Chara vulgaris*	0	0	10919	NC_008097
*Chlorokybus atmophyticus*	0	0	7640	NC_008822
*Mesostigma viride*	0	0	6056	NC_002186
*Staurastrum punctulatum*	No Inverted Repeat			NC_008116
*Zygnema circumcarinatum*	No Inverted Repeat			NC_008117

^†^Large repeats containing the ribosomal operon in chloroplast genomes were queried for size and sequence homology between the 16S and 23S ribosomal subunits. The three organisms *Cyanidioschyzon merolae (*Rhodophyta)*, Bryopsis hypnoides* (Chlorophyta) *and Monomorphina aenigmatica* (Chlorophyta) were omitted from the small repeat statistical analyses because of the presence of a greatly expanded tandem repeat structure or large (>200 bp) repeat structures, which causes an over representation the repeat structure due to the counting nature of the analyses presented.

*Cattolico RA, Jacobs M, Rocap G. unpublished genomic data.

Non-canonical ribosomal operon structure is rarely found (Additional file 4). Within haptophytes, *E. huxleyi* has a canonical ribosomal operon structure, but *P. antarctica* and *P. globosa* do not. In both *P. antarctica* and *P. globosa*, one copy of the ribosomal operon contains both tRNAs in the intergenic spacer region (conventional arrangement), while the second copy lacks both tRNAs in the intergenic spacer domain (non-conventional). As also seen in Figure 4, copies of the ribosomal gene sequences encoded within the *C. tobin* repeat, contain single nucleotide polymorphisms in the 16S (6 SNPs) and 23S (5 SNPs) ribosomal genes. Notably, every cryptophyte and rhodophyte chloroplast genome examined that encodes a repeat structure also shows the presence of SNPs between replicated ribosomal genes (Table 3). In contrast, only one alga in the chlorophyte lineage, *Ostreococcus tauri*, contains a SNP. Additionally, no land plant species queried show SNPs in either 16S or 23S genes. Though speculative, the presence of alternative operon structure for the ribosomal genes, combined with the elevated appearance of SNPs suggest that the well-recognized “copy correction” mechanism [42] may be more effective in some “green” algal lineages (chlorophytic algae and algae with chlorophytic algal symbionts), than in the “red” lineage of autotrophs (rhodophytes and CASH taxa). The route to repeat loss within an algal chloroplast genome may be augmented by the accumulation of SNPs and disintegration of operon integrity.

#### Small repeat function in chloroplast genomes

Chloroplast genomes are consistently embellished with small repeats that are either tandem or inverted in orientation. *Chrysochromulina tobin* is no exception having 16 inverted repeats with an average length of 25.4 +/− 5.2 bp in stem length with loop domains averaging 6.1 +/− 3.0 bp in size. A single tandem repeat comprised of a duplicated 15 bp sequence also occurs. Similar to observations made for other chloroplast genomes [43–46] and bacterial genomes [47–49], most *C. tobin* chloroplast repeats occur within the intergenic space, at the termini of genes located on opposite coding strands (Figure 2).

The conservation of repeats within the chloroplast genomes of all algal taxa suggests a functional constraint for these structures. Insight into the contribution of repeats to chloroplast metabolic processes was accomplished by elegant studies with *Chlamydomonas reinhardtii*[50–52] that exploited the early ability to transform this organism. Though these studies were predominantly focused on inverted repeats and confined to a limited gene set (e.g., *atp*B and *rbc*L), observations reveal a multifunctional role for repeat structures [53]. Data show that the presence of a repeat at the terminus of a gene is necessary for proper mRNA processing by exo- and endonucleases [54], maintaining RNA stability [52, 55] and supporting protein translation [50].

**Figure 5:**
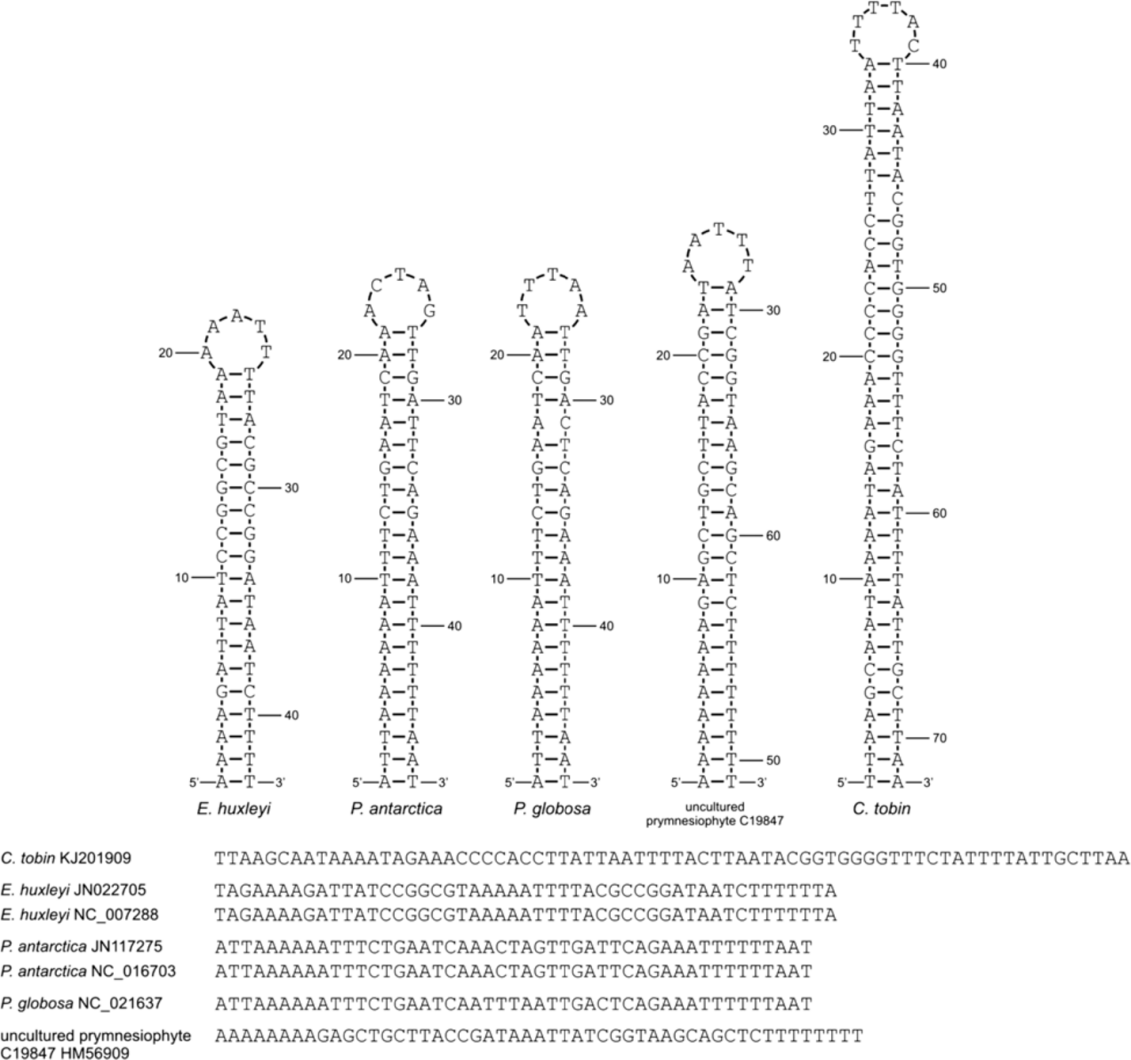
**Conserved inverted repeats found adjacent to haptophyte** ***clp*** **C genes.**

Given the established functional contribution of repeats, we asked whether specific genes or conserved chloroplast gene clusters [41] were targeted for repeat association. Significant gene-repeat association was observed. For example, an inverted repeat is found at the terminus (*rps*10) of the ribosomal protein operon that encompasses 24 genes; inverted repeats are often present after the RuBisCO operon (*rbc*L/*rbc*S), as well as following the photosystem II gene pair (*psb*C/*psb*D). Each of these targeted genes has no spatial relationship to one another with respect to in-chromosome placement; no concurrence exists among these genes in repeat type (tandem, inverted) and no similarity in sequence identity is seen in the repeat structures that are associated with the targeted gene. Despite these facts, selected gene-repeat associations (e.g., *rbc*L/*rbc*S) are conserved in chloroplast genomes as taxonomically disparate as *C. tobin and E. huxleyi* (Haptophyta), *Ectocarpus siliculosus* (Stramenopila: Phaeophyceae), *Cyanidium caldarium* (Rhodophyta) and *Rhodomonas salina* (Cryptophyta) - a list that is by no means exhaustive. Single genes that are not associated with operons can also be targeted for repeat tagging. A good example is *clp*C that encodes an ATPase-dependent protease. This gene is found in different locations with many dissimilar up and downstream gene neighbors among CASH taxa. *clp*C is tagged with a repeat in all haptophytes except *P. lutheri*. A repeat is also found next to *clpC* in 19 of 44 (43.2%) CASH plastid genomes analyzed to date. As shown in Figure 5, even within haptophytes, the repeat is conserved *only* in gene association. Neither the size, sequence, nor stem loop structure formed by these repeats is conserved.

**Figure 6:**
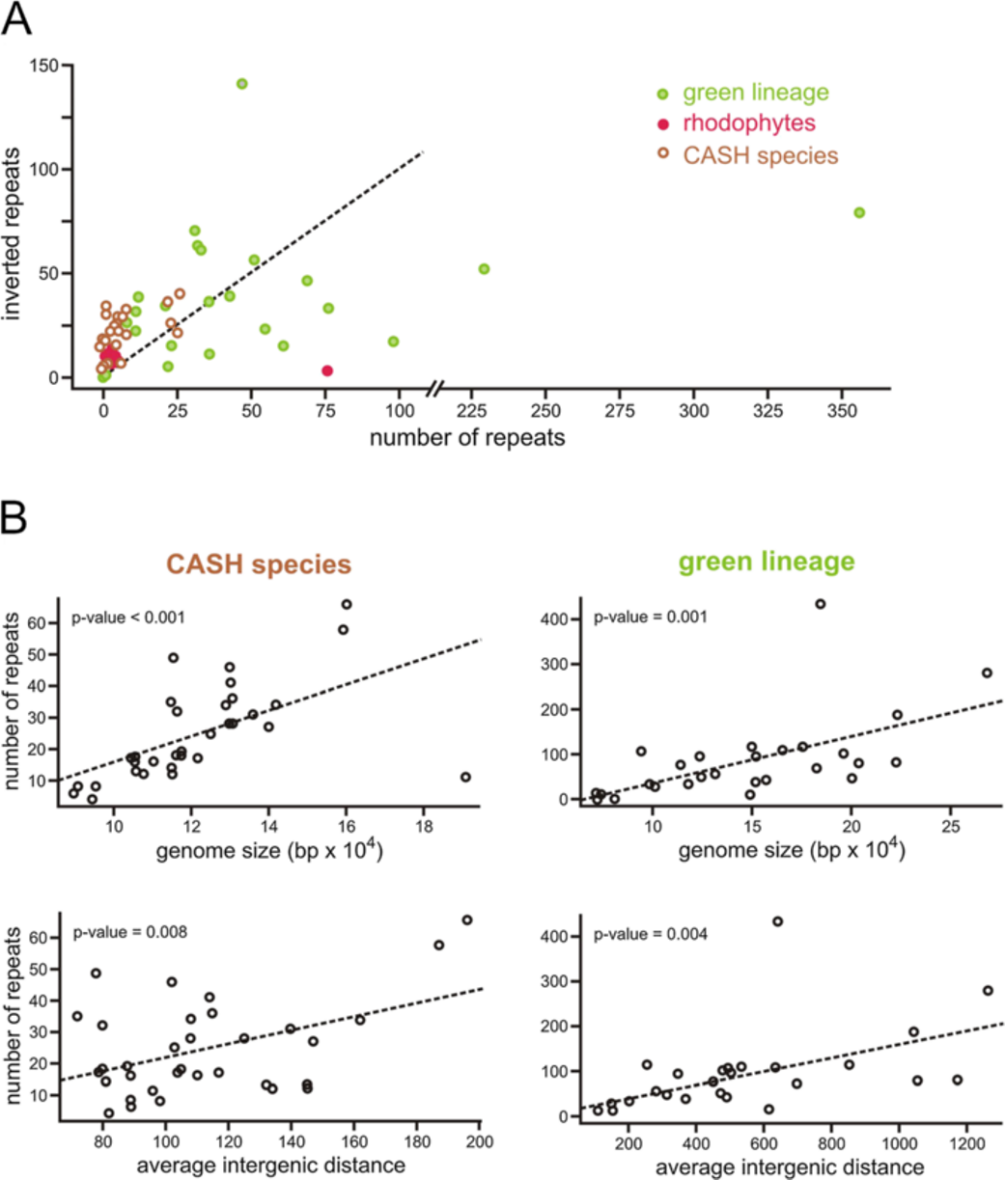
**Small repeat analysis across algal groups. (A)** Tandem and Inverted repeat complement across CASH, rhodophyte and “green” algal species. The dotted line represents a 1:1 ratio of tandem and inverted repeat counts. **(B)** Linear association of repeat number versus genome size and average intergenic distance.

Given that repeats appear to have a functional significance, it was also of interest to determine whether a pattern in repeat acquisition exists among evolutionary diverse algae. Repeat properties were queried across three groups of algae: rhodophytes (red algae), the ‘green’ algal lineage (green algae and those algae derived from the secondary endosymbiotic uptake of a chlorophyte [i.e. euglenids]) and the CASH grouping of algal species (derived from secondary or higher order endosymbioses of a rhodophytic plastid). Data clearly show that the number of repeats found in a chloroplast genome varies when different algal groups are compared. Rhodophytes appear to have few repeats (10 to 16 when excluding *Cyanidioschyzon merolae,* n = 79), and the CASH taxa have a moderate number (4 to 49 repeats [average = 25]). In contrast, the green plastid lineage has on average 80 repeats per genome, though representatives have as many as 281 (*Dunaliella salina*) to 435 (*Chara vulgaris*) (Additional file 2). Repeat type is also group dependent. CASH algae have a greater number of inverted repeats in their chloroplast genomes, whereas the green lineages have significantly more tandem repeats (Figure 6A). Attempts to assess whether differences in chloroplast size and intergenic distance influenced the number and size of repeat structures show both parameters to be positively correlated with an increase in repeat number for green and CASH plastid lineages (Figure 6B). However, there appears to be a limit on repeat size in the CASH plastid lineage, for even as genomes become larger and/or intergenic distances increase (Additional file 5), repeat size does not exceed ~65 bp. This result significantly contrasts with that seen in the green lineage. A strong correlation exists between increased repeat size, and either an increased genome size or an increased intergenic distance (Additional file 5). The fact that repeat embellishment occurs in every algal chloroplast genome analyzed to date, that repeats often are conserved near specific genes, and repeats contribute to chloroplast gene expression, suggest that future research analyzing chloroplast intergenic regions is warranted.

### Chloroplast protein characterization

Analysis of select genes can provide unique insight into chloroplast genome evolution and function. In this context, several genes within the *Chrysochromulina tobin* chloroplast genome are described below.

### Ribosomal protein RPL36

**Figure 7:**
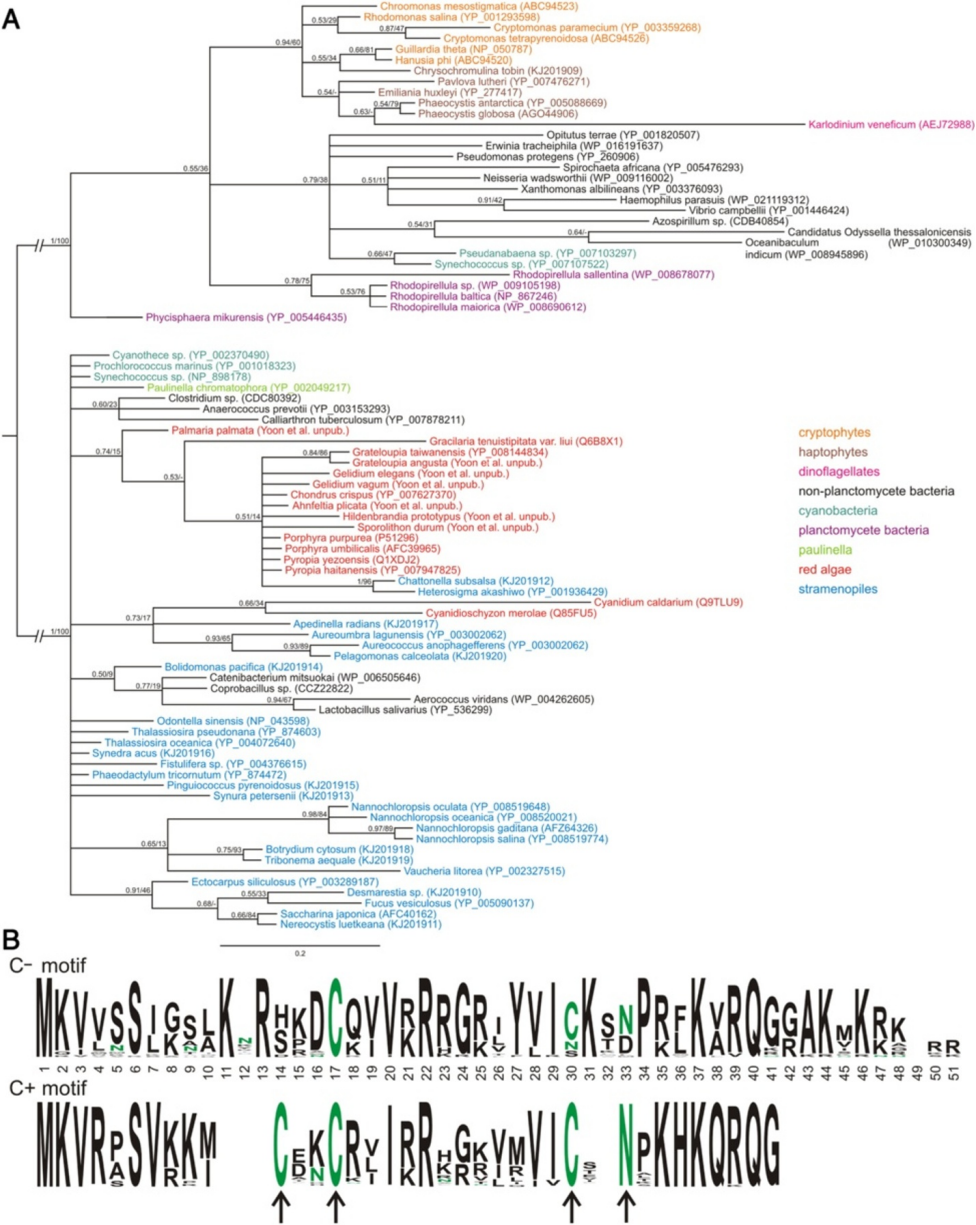
**Phylogenetic analysis of RPL36 proteins.** Bayesian majority rule consensus tree of 85 RPL36 proteins from haptophytes, cryptophytes, a haptophyte plastid-containing dinoflagellate, stramenopiles, rhodophytes, *Paulinella chromatophora*, and select bacteria and cyanobacteria*.* Taxa are colored according to the legend. Bayesian posterior probabilities and Maximum Likelihood bootstrap support are shown at nodes. Scale bar shows amino acid substitutions per site **(A)**. Logo plot consensus sequences for the C^−^ and C^+^ RPL36 protein **(B)**. The zinc finger residues are completely conserved in the C^+^ genotype, while 2 residues are absent from the C^−^ clade.

It is now known that some ribosomal proteins are multifunctional. Not only do these proteins serve as architectural components in the ribosome itself, but may also have additional extra-ribosomal functions that help maintain cellular homeostasis [56]. As shown in Figure 7B, the ‘conventional’ (C^+^ motif) RPL36 protein encoded by chlorophytes and rhodophytes has a highly conserved zinc finger motif of the cysteine-cysteine-cysteine-histidine (CCCH) type (indicated by arrows). The haptophyte and cryptophyte RPL36 (C^−^ motif) proteins lack the conserved zinc finger domain. In both haptophytes and cryptophytes the first cysteine is replaced by a serine, and the terminal histidine of the zinc finger motif is replaced by a leucine. Therefore it is very unlikely that the haptophyte/cryptophyte RPL36 C^−^ retains zinc finger protein function.

Although the zinc finger function was not recognized, earlier studies used the unique RPL36 C^−^ sequence observed in haptophyte and cryptophyte chloroplast genomes to argue for a sister relationship between the plastids of these taxa [57]. Sanchez-Puerta and Delwiche attempted to reconcile the presence of an *rpl*36 C^−^ gene (likely derived from a bacterium via lateral gene transfer) in the cryptophyte and haptophyte plastids with the presence of the ancestral *rpl*36 C^+^ gene in stramenopiles by positing that, for a time, two chloroplast genomes co-existed in the haptophytes and cryptophytes, some genomes containing the *rpl*36 C^+^ gene and others with the C^−^ gene [14]. One or the other genome was then fixed in particular lineages. This hypothesis predicts the discovery of an *rpl*36 C^+^ gene in the chloroplast genomes of some haptophytes or cryptophytes. To better test this hypothesis, we infer a new *rpl36* phylogeny including five additional haptophyte genera (seven species), 24 additional stramenopiles, four additional rhodophytes, as well as a representative of the recently recognized algal lineage *Paulinella*.

Mining NCBI as well as our publically available chloroplast genome database [38], a total of 462 non-redundant RPL36 amino acid sequences were recovered for phylogenetic analysis (Additional files 6, 7, 8 and 9). In Figure 7, this large dataset is condensed and re-inferred to include sequences from a limited number of bacterial and cyanobacterial representatives; rhodophytic as well as CASH plastids. We confirm the RPL36 C^−^ identity of all haptophyte and cryptophyte algae to the exclusion of all other eukaryotic algal taxa sampled, including stramenopiles and rhodophytes [57], thus Sanchez-Puerta and Delwiche’s hypothesis was not confirmed. Furthermore, even though our *rpl*36 phylogeny increases bacterial sampling beyond that used for prior analyses, results do not support a planctomycete-origin of the laterally transferred *rpl*36 as previously suggested [57]. No bacterial clade is strongly supported as the donor of the C^−^ gene (Additional files 6 and 7). Determination of the donor lineage is made difficult by the short length of the *rpl*36 protein (C^−^ 49 amino acids, C^+^ 38 amino acids) and the ancient nature of the lateral gene transfer event.

The duality in chloroplast encoded *rpl*36 genes poses questions concerning the contribution of each alternative protein type to the maintenance of cellular stasis in different algal lineages. The functional contribution is most likely multifaceted. Certainly, the RPL36 protein, whether of the C^+^ or C^−^ type, contributes to ribosomal structure [58]. However, bacteria that contain both paralogs of the RPL36 protein differentially express *rpl*36 C^−^ and *rpl*36 C^+^ when subject to zinc stress [59, 60]. The *rpl*36 C^−^ gene is up-regulated under limiting conditions. Since the zinc finger domain of RPL36 not only binds zinc, but also bind other cationic species [61], one might speculate that in algal cells an increased covalent ion binding potential might provide a competitive advantage when living in ecosystems where particular cofactors are in short supply. The fact that haptophyte/cryptophyte RPL36 C^−^ proteins have an extended 7 to 9 amino acid C terminus that is enriched with positively charged as well as hydrophobic moieties supports the possibility that this small molecule also has a regulatory function, similar to that established for other ribosomal zinc finger proteins [62–64].

### Two component signal transduction systems

Two-component regulatory systems are key mechanisms through which many organisms (bacteria, archaea, and eukaryotes) control responses to fluctuating environmental conditions [65, 66]. Numerous two component regulatory systems exist. In its simplest form, when cued by an external stimulus, a phosphoryl group from a conserved histidine residue within a sensor kinase protein is transferred to an aspartic acid in the receiver domain of a response regulator protein. Phosphorylation of the response regulator protein activates an effector domain (usually through a conformation change) to propagate the intended regulatory effect.

The *Chrysochromulina tobin* chloroplast genome encodes two response regulator proteins but is devoid of sensor kinase genes. The first response regulator protein, *orf*27, encodes a protein similar to the TRG1 response regulator described for the stramenopile *Heterosigma akashiwo*[41, 67] and is also found in other CASH species, rhodophytes, and cyanobacteria. The second response regulator, *ycf*55, is likely a member of a new subclass of response regulators evolved from the cyanobacterial type “CheY-like” response regulator proteins. Many of the cyanobacterial CheY-like homologs are comprised of approximately 550 amino acids and contain two domains; the aforementioned receiver domain and a conserved domain of unknown function (DUF3685), hypothesized to be the effector. Intriguingly, the *che*Y-like homolog (*ycf*55) found in *C. tobin* is comprised of only 314 amino acids. Multi-sequence protein alignments of a variety of response regulators from cyanobacteria, algae, and *Arabidopsis* revealed that the C-terminus of the *C. tobin ycf*55 is most similar to the cyanobacterial type CheY-like proteins, as both contain the terminal DUF3685 domain. In contrast, the N-terminus of the *C. tobin ycf*55 is divergent from both cyanobacterial CheY-like response regulators and the plant type response regulators (i.e., ARR1-14), including loss of the canonical site of phosphorylation. Nevertheless, sequences that resemble the *C. tobin* type of *ycf*55 are conserved in rhodophytes (*Chondrus crispus*, *Calliarthron tuberculosum*, *Gracilaria tenuistipitata*, *Porphyra purpurea*, and two *Pyropias* species), other haptophytes (except *P. lutheri*), and some cyanobacteria (classified as ‘RRI-other’ [68]) indicating that this protein still provides an important function. Within this divergent subclass of *ycf*55 proteins an aspartic acid residue (D43) just upstream of the canonical position is conserved, suggesting that this residue could replace the canonical site of phosphorylation by an as yet unknown sensor kinase.

### Structural analysis of Ycf39

**Figure 8:**
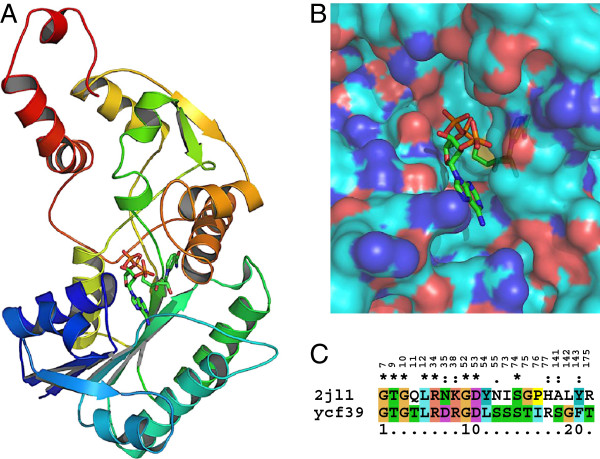
**Insights into 3D structure of ycf39 and its NADP binding potential. (A)** 3D model of ycf39 sequence based on comparative modeling using a crystal structure (PDB code 2JL1) as a template. **(B)** Amplified view of the cleft in ycf39 model and docked NADP molecule. **(C)** NADP binding residues compared between ycf39 and 2JL1. The numbers (top) represent the amino acid position in the template structure, 2JL1. The corresponding residues in ycf39 were found by multiple sequence alignment using HHpred server [74].

Ycf39 is conserved in many CASH and rhodophytic species. To gain insight into Ycf39 identity and its potential functional role, a structural and comparative modeling approach was taken. The HHpred server [69] was used to identify structures in the Protein Data Bank (PDB) [70] that showed high sequence homology to Ycf39. The top 10 structures recovered (Additional file 10) had E-values ranging from 9E^−39^ to 2.5E^−36^. Amino acid sequence identity of Ycf39 to these structural hits ranged between 15-18%. All structural hits show very similar fold identities, and fall under NmrA-like or NAD-binding domains by Pfam classification [71]. Eight out of ten structural homologs are known to bind NADP/NAD (Additional file 10). Using the ten structures as templates for Rosetta comparative modeling [72], a total of 20,400 trajectories were assessed and clustered based on backbone RMSD. The top scoring comparative model of Ycf39 (Figure 8A) is based on the structural template 2JL1 a triphenylmethane reductase (Additional file 11). The 2JL1 and Ycf39 sequence alignment is shown in Additional file 12.

To determine whether the proposed model of Ycf39 could bind an NADP molecule, a large number of NADP conformers (n = 885, see Methods) were randomly docked into the largest pocket in the Ycf39 3D model. Of the resulting random docked protein-ligand conformations obtained (n = 7,000), the top binding energy conformation is presented in Figure 8B. The model showed an equivalent positioning of NADP in the proposed pocket of the Ycf39 3D model as was observed in the template structure (PDB code 2JL1 and Additional file 11). In both cases, the nicotinamide group is buried in the protein, while the adenine group is near the surface. Additionally, locating the NADP interacting amino acids on the template (2JL1) and finding corresponding amino acids in Ycf39 based on sequence alignment showed 38% sequence identity and another 24% similarity (Figure 8C). These amino acids were in close proximity to the docked NADP in our Ycf39 model (Additional file 13).

Taken together, the sequence similarity of the Ycf39 protein product with NmrA-like family proteins (Additional file 1), the presence of a dockable site for NADP in the predicted structure of Ycf39 (Figure 8A, B) and striking similarity of the NADP interacting motif with a known NADP binder (Figure 8C) suggests Ycf39 should be identified as an NmrA-like protein in *C. tobin*. A strong homology (45% amino acid identity) of Ycf39 to an NmrA family protein identified in the endosymbiotic cyanobacteria *Nostoc azollae* has also been found [73]. Characterized NmrA proteins function in the transcriptional regulation of genes important to nitrogen metabolite repression [74, 75], thus allowing a cell to access a preferred nitrogen source [76]. Functional studies will be required to determine if the protein product of ycf39 serves this metabolic roll.

### Membrane transporter/translocator proteins

The majority of chloroplast proteins are encoded in the nucleus. For this reason, translocators located in the chloroplast envelope are needed to facilitate import of the cytosolically produced proteins into the plastid. Chloroplasts of primary endosymbiotic origin require protein transport across two membranes. This process is facilitated by two complexes, the TOC and TIC translocons, located in the outer and inner chloroplast envelopes respectively [77–79]. In plastids of higher order endosymbiotic origin (such as *Chrysochromulina tobin*), protein transport is complicated by the presence of either three or four membranes surrounding the chloroplast [80, 81]. Nonetheless, Tic and Toc components have previously been found in algae containing more than two membranes [82, 83]. Here, we identify two potential Tic subunit genes (*tic*20 and *tic*22) encoded in the *C. tobin* chloroplast genome. Tic20 is a small membrane protein that is anchored by three helices in the membrane and has protrusions into the stroma. A Tic20 homolog is likely encoded by the *ycf*60 gene, a gene found exclusively in rhodophyte, haptophyte and stramenopile chloroplast genomes. Tic22, a second member of the inner membrane import complex, has poorly characterized function. We have identified *ycf*80 as a Tic22 translocator protein homolog. *ycf*80 homologs are found exclusively in the chloroplast genomes of the haptophytes *C. tobin*, *E. huxleyi*, *P. antarctica* and the uncultured prymnesiophyte C19847, as well as rhodophytes. The continued sequestering of genes encoding these two translocator components of the innermost membrane of both primary and secondary plastids (the putative plasma membrane of the original symbiont) suggests that evolutionary footprints of original transporter components still remain in select taxa.

BLAST homology searches for nuclear encoded translocator homologs gave mixed results when querying the draft nuclear genome of *C. tobin* (Hovde, et. al. in preparation)*. toc*159 is the only nuclear encoded Toc gene found in *C. tobin. toc*34, *toc*75 and *toc*64 are absent or are diverged so identification was compromised.

Nuclear genes with strong homology to *tic*110 and *tic*55 are found. This observation is consistent with data for the rhodophyte *C. merolae*[84]. Interestingly, *tic*40 is also found in *C. tobin,* but is absent in rhodophytic queries.

## Conclusions

The complete sequence of *Chrysochromulina tobin* mitochondrial and chloroplast genomes, representative of the ecologically important haptophyte prymnesiales B2 clade, have been determined and annotated. Within the mitochondrial genome, a large repeat structure consisting of ~9 kb was found along with a novel 457 amino acid open reading frame of unknown function. The large inverted repeats in the chloroplast genome contain a combination of novel of intergenic spacer region structures and SNP variants when rDNA-containing domains are compared, indicating the possible loss of a copy correction mechanism. Notably, no recombined structural isomers of the *C. tobin* chloroplast genome were found. Small repeats within intergenic regions of the chloroplast genome have taxon-specific evolutionary features. The embellishment of specific genes by repeats argues for a functional role in metabolism for these structures. Several genes found in *C. tobin* chloroplast that remain uncharacterized, yet conserved in other algal species were analyzed. They include: the ribosomal protein *rpl*36; a new two component signal transduction protein*;* the potential NmrA-like NADP-binding nitrogen regulator, and two chloroplast protein import genes.

## Methods

### Culture maintenance

*Chrysochromulina* strain CCMP291, acquired from The National Center for Marine Algae (NCMA) by the Cattolico laboratory in 2006, was designated as P3. These cultures were maintained in 250 mL Erlenmeyer flasks containing 100 ml of RAC-1, a proprietary fresh water medium. Flasks were plugged with silicone sponge stoppers (Bellco Glass, Vineland, NJ) and capped with a sterilizer bag (Propper Manufacturing, Long Island City, NY). Large volume experimental cultures for genomic DNA harvesting were maintained in 1.0 L of RAC-1 medium contained in 2.8 L large-mouth Fernbach flasks. These flasks were plugged with hand-rolled, #50 cheese cloth-covered cotton stoppers and covered with a #2 size Kraft bag (Paper Mart, Orange, CA). All cultures were maintained at 20°C on a 12 hour light:12 hour dark photoperiod under 100 μEm^−2^ s^−1^ light intensity using full spectrum T12 fluorescent light bulbs (Philips Electronics, Stamford, CT). No CO_2_ was provided and cultures were not agitated.

Bacterized cultures were treated in the following manner to minimize bacterial contamination: P3 cultures were subject to re-iterative cell sorting using flow cytometry. *Chrysochromulina tobin* cells were stained for identification using BODIPY 505/515 (4,4-difluoro-1,3,5,7-tetramethyl-4-bora-3a,4a-diaza-s-indacene; Invitrogen, Carlsbad, CA), a neutral lipid binding fluorophore. Approximately 10 stained cells were sorted into a single well of a 96 well plate containing 100 μl RAC-1 medium. Due to poor growth in the 96 well plate, well contents were transferred to 10 ml of RAC-1 medium in 50 ml plastic tissue culture flasks (Nunc, Roskilde, Denmark). This cell sorting process was carried out 4 times with the resulting culture being designated as P4. Cells obtained from reiterative flow cytometric selection (P4) were then treated in RAC-1 medium that contained either streptomycin (resulting in culture P5.5) or hygromycin (P5.6). Treatment with these two antibiotics were identical: cells were exposed to a final concentration of 400 μg/ml antibiotic for 18 hours before 5 mL of treated cultures were transferred to 100 mL of antibiotic free RAC-1 medium. Cultures P5.5 and P5.6 were periodically tested for bacterial contamination using liquid LB medium made with RAC-1 medium in replacement of water. Sequencing data and a cultured isolate has shown that one bacterial contaminant is still present in the antibiotic treated cultures.

### Genomic DNA isolation

Total genomic DNA was collected from each of the P5.5 and P5.6 cultures using the Qiagen Genomic-tip Maxi DNA extraction protocol (Germantown, MD) with the following changes to the standard protocol [41]: 1.5 × 10^8^ cells were harvested by centrifugation (Beckman-Coulter JA-10 Rotor at 7000 rpm (5378 × g) for 20 minutes) and resuspended in lysis buffer (20 mM EDTA, pH 8.0; 10 mM Tris-base, pH 8.0; 1% Triton X; 500 mM Guanidine; 200 mM NaCl) with 1.0 hour incubation at 37°C. RNase A was added to a final concentration of 200 μg/ml and incubated for 30 minutes at 37°C. 600 μl of Proteinase K (20 mg/ml) (Sigma-Aldrich) was then added to the tube and incubated at 50°C for 2.0 hours, mixing every 30 minutes by swirling. The Qiagen DNA binding tip (Maxi size) was equilibrated using the manufacturer’s instructions. DNA preparation was transferred into the tip and allowed to pass using gravity at room temperature. The tip was washed twice using Qiagen buffer QC. 15 ml of Buffer QF (at 37°C) was added to the tip to elute the DNA. DNA was precipitated by the addition of 10.5 ml of 100% room temperature isopropanol followed by centrifugation (12,000 rpm (11,220 × g) for 20 min, 4°C using a JA-20 rotor). The pellet was washed in 4 ml of 4°C 70% ethanol and centrifuged again using the same conditions. The DNA pellet was air dried for 5 min and resuspended in warmed Qiagen buffer EB (50°C) and incubated at 50°C for 2.0 hours. DNA solution was quantitated using a spectrophotometer and subsequently transferred to 1.7 ml Eppendorf tubes and stored at −80°C.

### Genome sequencing, assembly and annotation

The *Chrysochromulina tobin* chloroplast and mitochondrial genomes were sequenced using a combination of Illumina [85] and 454 sequencing technologies [22]. Two shotgun libraries (2 × 100 and 1 × 150 base pair) were prepared using standard TruSeq protocols and sequenced from bulk *C. tobin* genomic DNA on an Illumina HiSeq2000 sequencer. Additional shotgun single-end and paired-end (10 kb insert) DNA libraries were prepared for sequencing on the 454 Titanium platform generating 1.2 million and 3.5 million reads, respectively. The 454 single-end data and the 454 paired end data (insert size 8180 +/− 1495 bp) were assembled together using Newbler, version 2.3 (release 091027_1459). The Illumina-generated sequences were assembled separately with VELVET, version 1.0.13 [23]. The resulting consensus sequences from both the VELVET and Newbler assemblies were computationally shredded into 10 kb fragments and were re-assembled with reads from the 454 paired end library using parallelPhrap, version 1.080812 (High Performance Software, LLC). Based on homologous BLAST [86] searches against other chloroplast and mitochondrial genomes, the mitochondrial genome was identified as a single contig of 25,263 bp with one gap and the chloroplast genome was comprised of two contigs that totaled a combined 101,192 bp. Most mis-assemblies in the contigs of the mitochondrial and chloroplast genomes were corrected using gapResolution (Cliff Han, unpublished script, Los Alamos National Laboratory) or Dupfinisher [87]. However, due to the large ribosomal inverted repeat in the chloroplast, PCR amplification anchored by priming of unique regions flanking and within the repeat sections was used as sequence template to resolve the final circular representation of the chloroplast genome structure. Similarly, a large tandem repeat structure identified in the mitochondrial genome prevented automated closure of the remaining gap. De-convolution of this repeat was completed by PCR amplification and cloning of multiple products (see primer table Additional file 14) into the pGem T-easy vector (Promega, Madison, WI) followed by capillary sequencing. The presence of chloroplast genomes containing flip-flop recombined isoforms was queried using all combinations of single copy region primers (*petB*, *ycf60*, *psa* and *rpl21* primers) (Additional file 14). Only the expected primer pairs, *petB-ycf60* and *psa-rpl21* pairs, produced PCR products.

The final, fully assembled chloroplast and mitochondrial genomes were supported by > 500× average coverage from the combined sequencing platforms. Each assembled genome was verified by aligning the original Illumina reads to the final draft using the Burrow-Wheeler Alignment tool (BWA) [88]. Continuous coverage without gaps or missing reads was verified using Tablet alignment [89] which identified >10 single nucleotide mismatches (both SNPs and indels) in the chloroplast draft assembly, which were corrected in the final assembly.

Annotation was accomplished by GLIMMER [90] for initial gene calling. BLAST homology searches to CpBase, a curated chloroplast database housed by the University of Washington Department of Oceanography [38] were used for final gene identification and recovery of small or missing genes that were overlooked by automated annotation. Manual examination of BLAST homology searches was performed for each protein coding gene to determine correct start codons and gene length. An identical approach was used to assemble the mitochondrial genome.

### Comparative genomic analyses

For comparative analysis of gene, tRNA and repeat content, genome size and GC content, the chloroplast genome of *C. tobin* was assessed using CpBase. Visualization of chloroplast and mitochondrial genome gene order comparison was completed using Mauve [28] and GRIMM analysis was performed respectively [37]. Mauve analysis of the three haptophyte genomes was performed using default seed weight, iterative alignment, determine LCBs and sum of pairs LCB scoring settings. GRIMM was performed on the Mauve output using default GRIMM settings. Additionally, genome map images were constructed using GenomeVx [91].

*Small repeat analysis:* Small (<200 bp) inverted repeats were identified using Einverted from EMBOSS [92]. Tandem repeats were identified using Tandem Repeats Finder [93]. Small repeats located next to genes (*clp*C*, psa*B*, rpo*C*, atp*A*, rps*10*, rbc*S*,* and *psb*C*)* appearing to be conserved across chloroplast genomes in multiple species from manual inspection were quantified across all CASH taxa available in CpBase [38]. To refine the repeat list for the gene/repeat association analysis, the presence of tandem or inverted repeats adjacent to genes or gene clusters was queried using the “Repeat Finder” tool in CpBase [38]. Parameters used were: Search distance: 300 bp, End: “Both”, Boundary: “Both”. Additionally, no other feature (tRNA or protein coding gene) separating the gene and inverted repeat was counted in this analysis. Small inverted repeat physical structure was determined by inputting the sequence into M-fold [94–96] using default parameters and a loop size maximum of 30 bp.

*Large inverted repeat homology:* The size of large inverted repeats which contain at least the ribosomal 16S-23S operon was determined by 2 sequence BLAST comparison of the two halves of a genome, each half containing one of the ribosomal repeats. BLASTN homology using the default settings was used to determine the borders of the repeat regions. To determine the sequence homology between two copies of 16S or 23S, BLASTN was used with default parameters. Each SNP and single nucleotide insertion or deletion was counted separately. If only one copy of the ribosomal operon was present, no homology search was performed.

*Small repeat statistical analysis:* A Fisher’s Exact test was implemented to compare the proportion of chloroplast with more tandem repeats than inverted repeats in CASH taxa, rhodophytes, and the “green lineage”. Linear regressions were used to test for an association between small repeats, genome size, and intergenic length with average repeat size as well as total small repeats with genome size and intergenic length. This analysis was repeated on the green lineage, CASH algae, and rhodophytes.

### Phylogenetic analysis of *rpl*36

By mining CpBase as well as NCBI, a total of 462 non-redundant RPL36 amino acid sequences were recovered for phylogenetic analysis. These sequences were aligned in MUSCLE [97] and any C-terminal extensions were trimmed to create a 41 amino acid alignment with two gaps in the RPL36 C^−^ proteins such that functional motifs of the zinc finger domain (and their substituted amino acids in the C^−^ proteins) were aligned. Protein matrices available in the CIPRES Science Gateway [98] MrBayes 3.2.2 tool were evaluated for appropriateness using ProtTest 2.4 [99]. The cpREV + I + Γ model of protein sequence evolution was found to best suit the data. Gene trees were inferred with RAxML 7.6.3 [100]with 1000 bootstraps, as well as with MrBayes v3.2.2 [101] with two runs each of four chains, 10 million generations, and 25% burn-in. Stationarity and convergence of the Bayesian analysis were assessed with Tracer v1.5 [102]. To best represent the data, 85 select taxa were chosen for Figure 7 and the phylogeny re-inferred with the same parameters but only 5 million generations in the Bayesian analysis.

### Structural modeling of Ycf39

A structural model of Ycf39 was built using template structures found on HHpred server [69]. Secondary structure prediction for Ycf39 sequence was made using PsiPred [103] (Additional file 15). Robetta server [104] was used to generate the peptide fragments based on local homology of the ycf39 sequence with other sequences in a structural database. Rosetta comparative modeling protocol [72] uses a secondary structure profile of the query sequence, customized three- and nine- amino acid long peptide fragments based on secondary structure prediction of the query sequence from the structural database, a multiple sequence alignment file and a structural template to build the tertiary models of the query sequence. A total of 20440 comparative modeling trajectories were run and a few top scoring models visualized using PyMOL (The PyMOL Molecular Graphics System, Schrödinger, LLC).

To dock NADP to the top-ranked predicted ycf39 structures, NADP atomic coordinates were taken from one of the HHpred search hits (PDB code 2JL1) that had a bound NADP molecule. Protons were added to this NADP structure using Avogadro molecule editor software [105], while the phosphate groups were kept deprotonated and the nicotinamide group was kept planar to match the deprotonated form of the molecule. The formal charge of the resulting NADP molecule was zero. A PubChem [106] search identified 13 rotatable bonds in NADP that were sampled in two states, ± 30° from the dihedral angles observed in the 2JL1 crystal structure. The resulting library of 885 NADP conformers (Additional file 16) had full atom intra-molecule repulsive energies as calculated by Rosetta [107] within 1% of the starting molecule structure.

Random docking of NADP conformers to structural models of ycf39 was performed in three steps using the RosettaLigand protocol [108, 109]. NADP conformers were randomly placed in the largest cleft observed in ycf39 3D model, then rotated in 1000 random orientations to identify orientations with the best shape complementarity to the protein surface. Small perturbations in translation and rotation (0.1 Å, 3°) were introduced with side chain repacking and energy minimization, followed by a second round of small perturbations in NADP position, orientation and torsions prior to a final energy minimization. A total of 7000 trajectories were run using the above strategy to identify the best ycf39 structural model with a bound NADP molecule and the lowest ligand binding energy.

## Electronic supplementary material

Additional file 1: Figure S1: Mauve analysis of conserved regions in the mitochondrial genomes of *Emiliania huxleyi, Phaeosyctis antarctica*, and *Chrysochromulina tobin.* (PDF 344 KB)

Additional file 2: Table S1: Genome size, intergenic distance and repeats across algal species used in the statistical analysis section. (XLS 44 KB)

Additional file 3: Figure S2: Detailed intergenic spacer region comparison of the two *Chrysochromulina tobin* ribosomal operon repeats. (PDF 274 KB)

Additional file 4: Table S2: Other chloroplast genomes containing non-canonical ribosomal intergenic spacer regions. (PDF 304 KB)

Additional file 5: Figure S3: Additional repeat analysis of CASH and green algal lineages. (PDF 312 KB)

Additional file 6: **Bayesian RPL36 phylogenetic tree with complete dataset; open in FigTree.** (ZIP 31 KB)

Additional file 7: **RPL36 alignment with complete dataset; nexus formatted.** (ZIP 17 KB)

Additional file 8: **Bayesian RPL36 phylogenetic tree with reduced dataset (Figure** 7 **); open in Mesquite.** (ZIP 6 KB)

Additional file 9: **RPL36 alignment with reduced dataset; nexus formatted.** (ZIP 3 KB)

Additional file 10: Table S4: Top protein data bank structural hits to the NmrA protein (Ycf39) in the C. tobin chloroplast genome. (PDF 304 KB)

Additional file 11: Figure S4: Structural template (PDB code 2JL1) for comparative modeling of Ycf39 sequence and NADP in the binding pocket of protein. (PDF 312 KB)

Additional file 12: Figure S5: Multiple sequence alignment of ycf39 and one of the templates (PDB code 2JL1) used for comparative modeling. (PDF 477 KB)

Additional file 13: Figure S6: NADP interacting residues in 2JL1 and Ycf39 models. (PDF 570 KB)

Additional file 14: Table S5: Primers used for organellar genome resolution of repeat structures. (PDF 291 KB)

Additional file 15: Figure S7: Secondary structure prediction of Ycf39 sequence using Psipred. (PDF 243 KB)

Additional file 16: Figure S8: Conformers of NADP molecule used for RosettaLigand Dock protocol. (PDF 339 KB)

## Abbreviations

ISR: Intergenic Spacer Region
SNP: Single Nucleotide Polymorphism
RMSD: Root Mean Square Deviation
NADP: Nicotinamide Adenine Dinucleotide Phosphate.
