# Supplementary material for: The mitochondrial and chloroplast genomes of the haptophyte Chrysochromulina tobin contain unique repeat structures and gene profiles
Source: BMC Genomics. 2014 Jul 17;15:604. doi: 10.1186/1471-2164-15-604 (PMC4226036; doi:10.1186/1471-2164-15-604)
Supplement: Supplementary file 1 — Additional file 1: Figure S1: Mauve analysis of conserved regions in the mitochondrial genomes of Emiliania huxleyi, Phaeosyctis antarctica, and Chrysochromulina tobin. (PDF 344 KB) [file 12864_2014_7065_MOESM1_ESM.pdf]

Additional File 1:

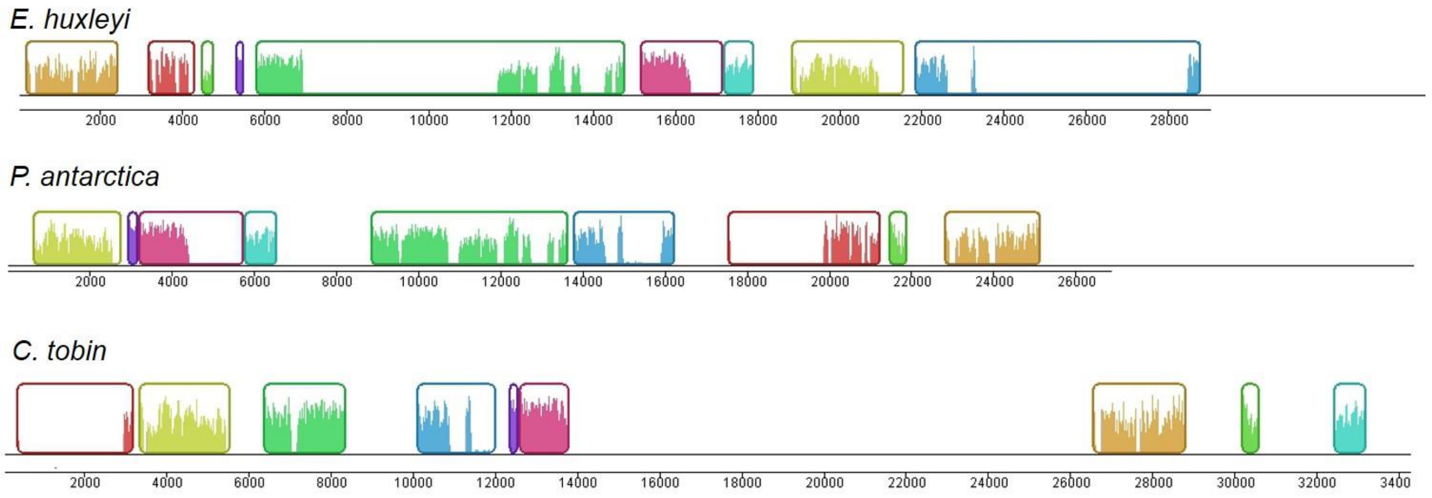

Supplementary figure 1: Mauve analysis of conserved regions in the mitochondrial genomes of *Emiliania huxleyi, Phaeocystis antarctica*, and *Chrysochromulina tobin.*
