# Supplementary material for: The mitochondrial and chloroplast genomes of the haptophyte Chrysochromulina tobin contain unique repeat structures and gene profiles
Source: BMC Genomics. 2014 Jul 17;15:604. doi: 10.1186/1471-2164-15-604 (PMC4226036; doi:10.1186/1471-2164-15-604)
Supplement: Supplementary file 3 — Additional file 3: Figure S2: Detailed intergenic spacer region comparison of the two Chrysochromulina tobin ribosomal operon repeats. (PDF 274 KB) [file 12864_2014_7065_MOESM3_ESM.pdf]

Additional file 3:

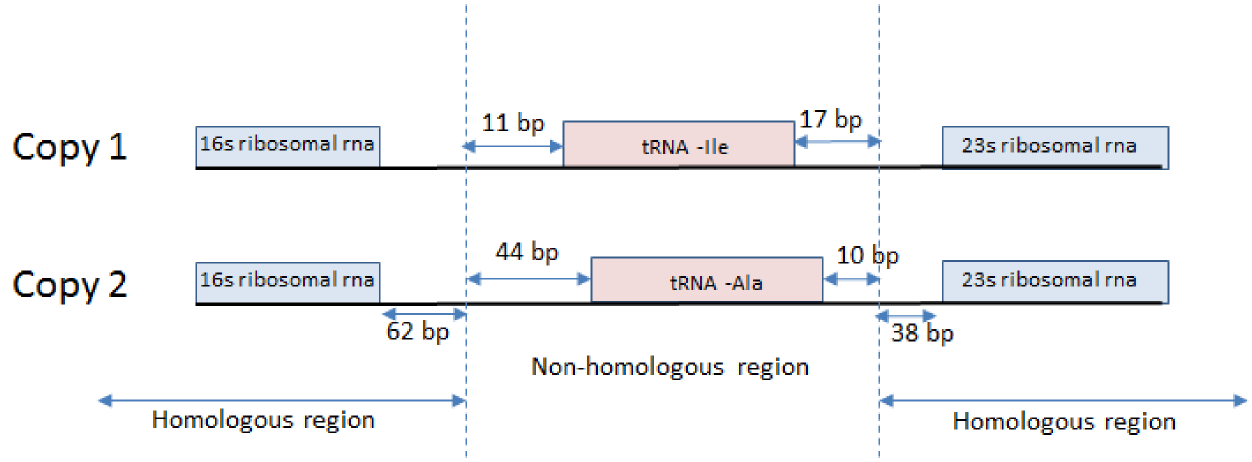

Supplementary figure 2: Detailed intergenic spacer region comparison of the two *Chrysochromulina tobin* ribosomal operon repeats.
