# Supplementary material for: The mitochondrial and chloroplast genomes of the haptophyte Chrysochromulina tobin contain unique repeat structures and gene profiles
Source: BMC Genomics. 2014 Jul 17;15:604. doi: 10.1186/1471-2164-15-604 (PMC4226036; doi:10.1186/1471-2164-15-604)
Supplement: Supplementary file 4 — Additional file 4: Table S2: Other chloroplast genomes containing non-canonical ribosomal intergenic spacer regions. (PDF 304 KB) [file 12864_2014_7065_MOESM4_ESM.pdf]

Additional File 10:

Table 4: **Top structural hits to the NmrA protein (Ycf39) in the *C. tobin* chloroplast genome**

PDB ID	Name	Pfam classification*	NADP / NAD binding?
2JL1	Triphenylmethane reductase	NmrA-like family	Yes (NADP)
3E48	Nucleoside-diphosphate-sugar epimerase	NmrA-like family	No (Mg)
3I6I	Putative leucoanthocyanidin reductase 1	NmrA-like family	Yes (NADP)
2X4G	Nucleoside-diphosphate-sugar epimerase	NADH(P)-binding	Yes (?)
3M2P	UDP-N-acetylglucosamine 4-epimerase	NAD dependent epimerase	No (UDP)
2ZCU	NADPH-dependent quinone oxidoreductase (QOR2)	NmrA-like family	Yes (?)
2WM3	NmrA-like family domain containing protein 1 in complex with niflumic acid	NmrA-like family	Yes (NADP)
4ID9	Short-chain dehydrogenase/reductase superfamily protein	NAD dependent epimerase	Yes (NAD)
3RUF	Unnamed	NAD dependent epimerase	Yes (NAD)
2C5A	GDP-mannose-3', 5' -epimerase	NAD dependent epimerase	Yes (NAD)

*: Some proteins fall under more than one Pfam description, but we only mention NmrA/NAD related families
