# Supplementary material for: The mitochondrial and chloroplast genomes of the haptophyte Chrysochromulina tobin contain unique repeat structures and gene profiles
Source: BMC Genomics. 2014 Jul 17;15:604. doi: 10.1186/1471-2164-15-604 (PMC4226036; doi:10.1186/1471-2164-15-604)
Supplement: Supplementary file 5 — Additional file 5: Figure S3: Additional repeat analysis of CASH and green algal lineages. (PDF 312 KB) [file 12864_2014_7065_MOESM5_ESM.pdf]

Additional File 11:

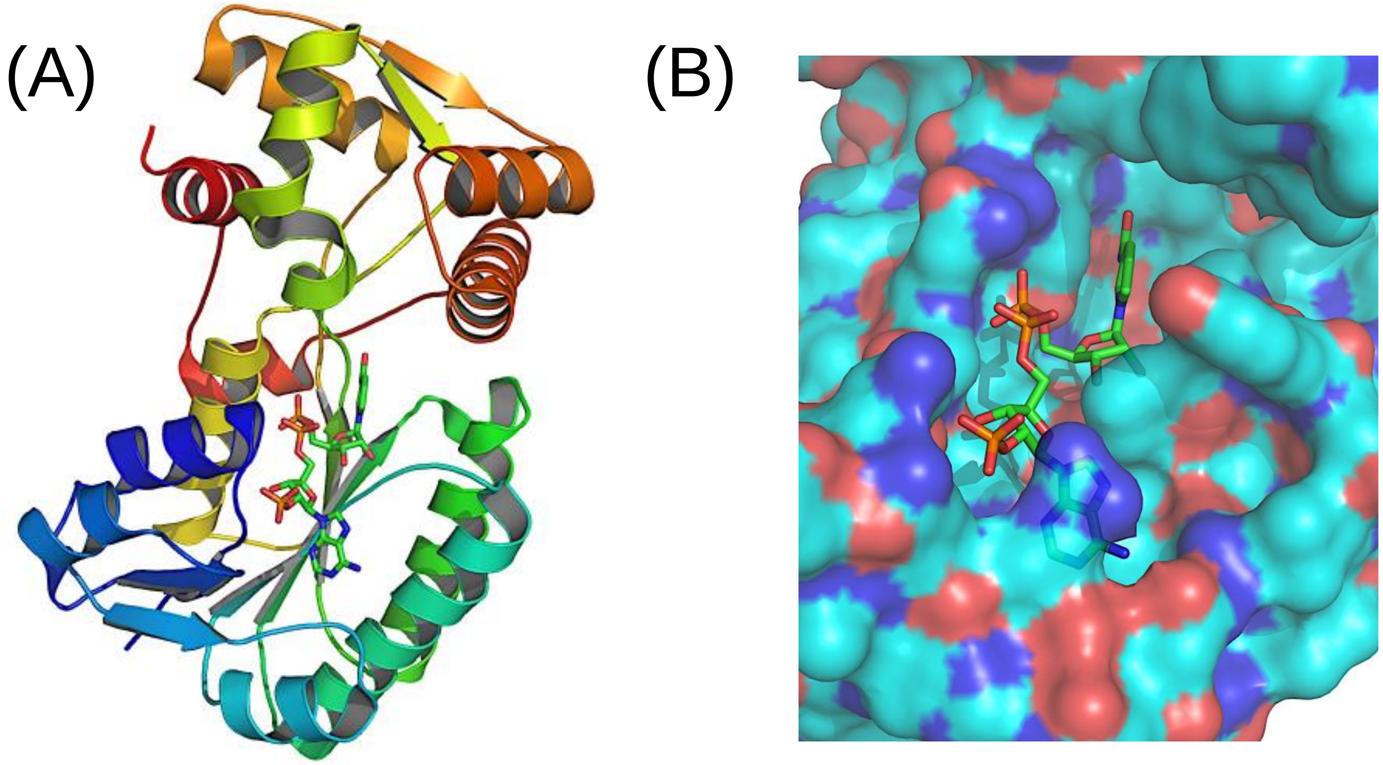

Supplementary Figure 4: (A) Structural template (PDB code 2JL1) for comparative modeling of ycf39 sequence. (B) NADP (shown as sticks) in the binding pocket of protein (shown as solid surface)
