# Supplementary material for: The mitochondrial and chloroplast genomes of the haptophyte Chrysochromulina tobin contain unique repeat structures and gene profiles
Source: BMC Genomics. 2014 Jul 17;15:604. doi: 10.1186/1471-2164-15-604 (PMC4226036; doi:10.1186/1471-2164-15-604)
Supplement: Supplementary file 12 — Additional file 12: Figure S5: Multiple sequence alignment of ycf39 and one of the templates (PDB code 2JL1) used for comparative modeling. (PDF 477 KB) [file 12864_2014_7065_MOESM12_ESM.pdf]

Additional file 12:

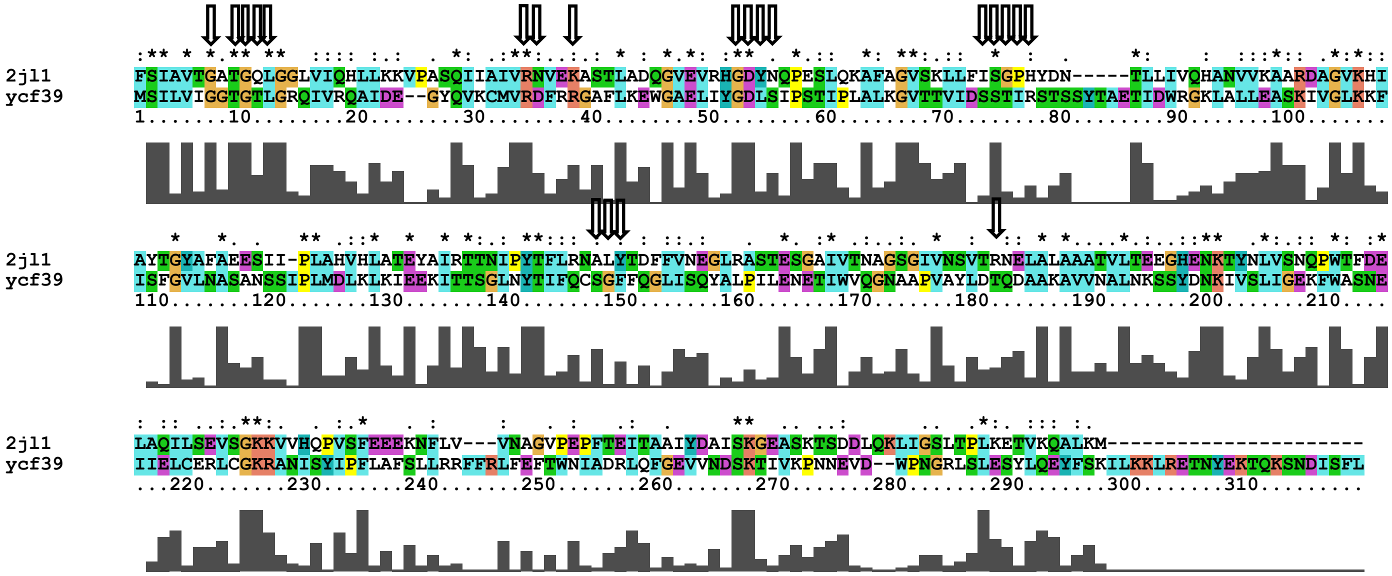

Supplementary Figure 5: Multiple sequence alignment of ycf39 and one of the templates (PDB code 2JL1) used for comparative modeling. The alignment was carried by HHpred server [1] and figure was created using ClustalX software [2]. The identical and similar residues are maked with '*'. ':' and '.' where the later two shows different level of similarities. Identical and similar positions are also represented by the height of the column beneath each residue. The residue positions marked with arrow are NADP binding residues in 2JL1.

1. Söding J, Biegert A, Lupas AN: The HHpred interactive server for protein homology detection and structure prediction. *Nucleic Acids Res* 2005, 33(Web Server issue):W244–248.

2. Larkin MA, Blackshields G, Brown NP, Chenna R, McGettigan PA, McWilliam H, Valentin F, Wallace IM, Wilm A, Lopez R, Thompson JD, Gibson TJ, Higgins DG: Clustal W and Clustal X version 2.0. *Bioinforma Oxf Engl* 2007, 23:2947–2948.
