# Supplementary material for: The mitochondrial and chloroplast genomes of the haptophyte Chrysochromulina tobin contain unique repeat structures and gene profiles
Source: BMC Genomics. 2014 Jul 17;15:604. doi: 10.1186/1471-2164-15-604 (PMC4226036; doi:10.1186/1471-2164-15-604)
Supplement: Supplementary file 13 — Additional file 13: Figure S6: NADP interacting residues in 2JL1 and Ycf39 models. (PDF 570 KB) [file 12864_2014_7065_MOESM13_ESM.pdf]

Additional File 13:

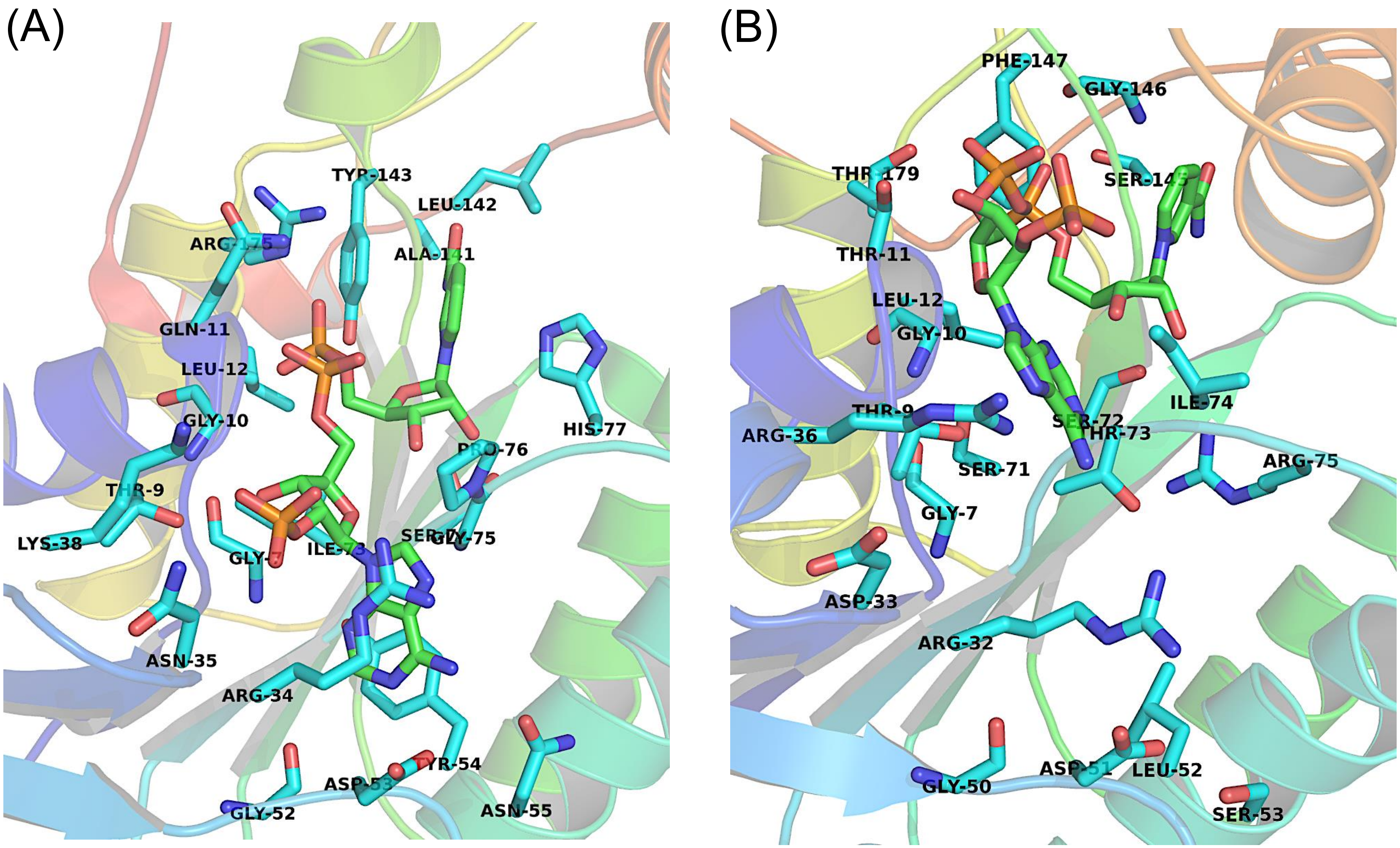

Supplementary Figure 6. NADP interacting residues. (A) Template PDB 2JL1. (B) Comparative model of ycf39 . A 3D representation of the amino acid sequences shown in Figure 9C.
