# Supplementary material for: The mitochondrial and chloroplast genomes of the haptophyte Chrysochromulina tobin contain unique repeat structures and gene profiles
Source: BMC Genomics. 2014 Jul 17;15:604. doi: 10.1186/1471-2164-15-604 (PMC4226036; doi:10.1186/1471-2164-15-604)
Supplement: Supplementary file 14 — Additional file 14: Table S5: Primers used for organellar genome resolution of repeat structures. (PDF 291 KB) [file 12864_2014_7065_MOESM14_ESM.pdf]

Additional File 14:
Supplementary table 5: Primers used for organellar genome resolution of repeat structures

**Mitochondrial genome finishing primers**	
5' Large tandem repeat F	GTTGGTCAAAAATAAGCAGAC
3' Large tandem repeat #1 F	TTGCTTCGGTCTTTCGCGTTC
3' Large tandem repeat #2a F	CATAAGTAAATTTCCTTTGATC
Flanking repeat region 1 F	CCGTTTTTAATGTTATTTTTTTC
cox1 anchored primer F	GGA TTT TCC AGA CGC TTA CAC
ORF457 anchored primer R	CACTGGAAACAGTCTCTACAA
**Chloroplast genome finishing primers**	
internal 23S primer F	CGGATACCTTGGTGTTCAGAAGC
internal 23S primer R	GCTTCTGAACACCAAGGTATCCG
petB anchored primer F (IR-A)	CTTGCGGACCATCTGTGAACAG
ycf60 anchored primer R (IR-A)	CTTTTCTGTTAGGGTTTCGCGAC
psa anchored primer F (IR-B)	CACTTTTCAAATGACGTGGCAAC
psa anchored primer 2 F (IR-B)	CTATCAAAGTCATGTGCATCAGC
rpl21 anchored primer R (IR-B)	CTTATCACCTTTGTCTAACGGTAG
