# Supplementary material for: The mitochondrial and chloroplast genomes of the haptophyte Chrysochromulina tobin contain unique repeat structures and gene profiles
Source: BMC Genomics. 2014 Jul 17;15:604. doi: 10.1186/1471-2164-15-604 (PMC4226036; doi:10.1186/1471-2164-15-604)
Supplement: Supplementary file 15 — Additional file 15: Figure S7: Secondary structure prediction of Ycf39 sequence using Psipred. (PDF 243 KB) [file 12864_2014_7065_MOESM15_ESM.pdf]

Additional File 15:

```
Conf: 979998898779999999999789979999658565444035896799954677466899
Pred: CEEEEECCCCHHHHHHHHHHHHCCCEEEEEECCHHHHHHHCCCCCEEEEEECCCHHHHHH
  AA: MSILVIGGTGTLGRQIVRQAIDEGYQVKCMVRDFRRGAFLKEWGAELIYGDLSIPSTIPL
              10        20        30        40        50        60


Conf: 963785765411025787531034568888877888864355643776245566799981
Pred: HHCCCCEEEEECCCCCCCCCCHHHHHHHHHHHHHHHHHHCCCCCEEEECCCCCCCCCCCC
  AA: ALKGVTTVIDSSTIRSTSSYTAETIDWRGKLALLEASKIVGLKKFISFGVLNASANSSIP
              70        80        90       100       110       120


Conf: 245566999999872544231354101366326555444327853426899743834499
Pred: HHHHHHHHHHHHHHHCCCCEECCCCCCCCCCHHHHHHHHHCCCCEEECCCCCCCCCEEHH
  AA: LMDLKLKIEEKITTSGLNYTIFQCSGFFQGLISQYALPILENETIWVQGNAAPVAYLDTQ
             130       140       150       160       170       180


Conf: 999999999709565795899718985589999999999978998779889899999999
Pred: HHHHHHHHHHHCCCCCCCEEEEECCCCCCHHHHHHHHHHHHCCCCCEEECCHHHHHHHHH
  AA: DAAKAVVNALNKSSYDNKIVSLIGEKFWASNEIIELCERLCGKRANISYIPFLAFSLLRR
             190       200       210       220       230       240


Conf: 874245433089999988875478700476021046778615999999999999999986
Pred: HHHHCCCCHHHHHHHHHHHHHCCCCCCCCCCCCCCCCCCCCCHHHHHHHHHHHHHHHHHH
  AA: FFRLFEFTWNIADRLQFGEVVNDSKTIVKPNNEVDWPNGRLSLESYLQEYFSKILKKLRE
             250       260       270       280       290       300


Conf: 176520111166439
Pred: CCCCCHHHHCCCCCC
  AA: TNYEKTQKSNDISFL
             310
```

Supplementary Figure 7: Secondary structure prediction of ycf39 sequence using Psipred (Jones 1999). Top row or 'Conf' shows confidence in prediction (9 being highest and 0 being lowest). Middle row or 'Pred' is the secondary structure predicted, 'H' for helix, 'E' for strand and 'C' for coil/loop. Third row, 'AA' is the amino acid identity at each position in ycf39 sequence.
