# Supplementary material for: The mitochondrial and chloroplast genomes of the haptophyte Chrysochromulina tobin contain unique repeat structures and gene profiles
Source: BMC Genomics. 2014 Jul 17;15:604. doi: 10.1186/1471-2164-15-604 (PMC4226036; doi:10.1186/1471-2164-15-604)
Supplement: Supplementary file 16 — Additional file 16: Figure S8: Conformers of NADP molecule used for RosettaLigand Dock protocol. (PDF 339 KB) [file 12864_2014_7065_MOESM16_ESM.pdf]

Additional File 16:

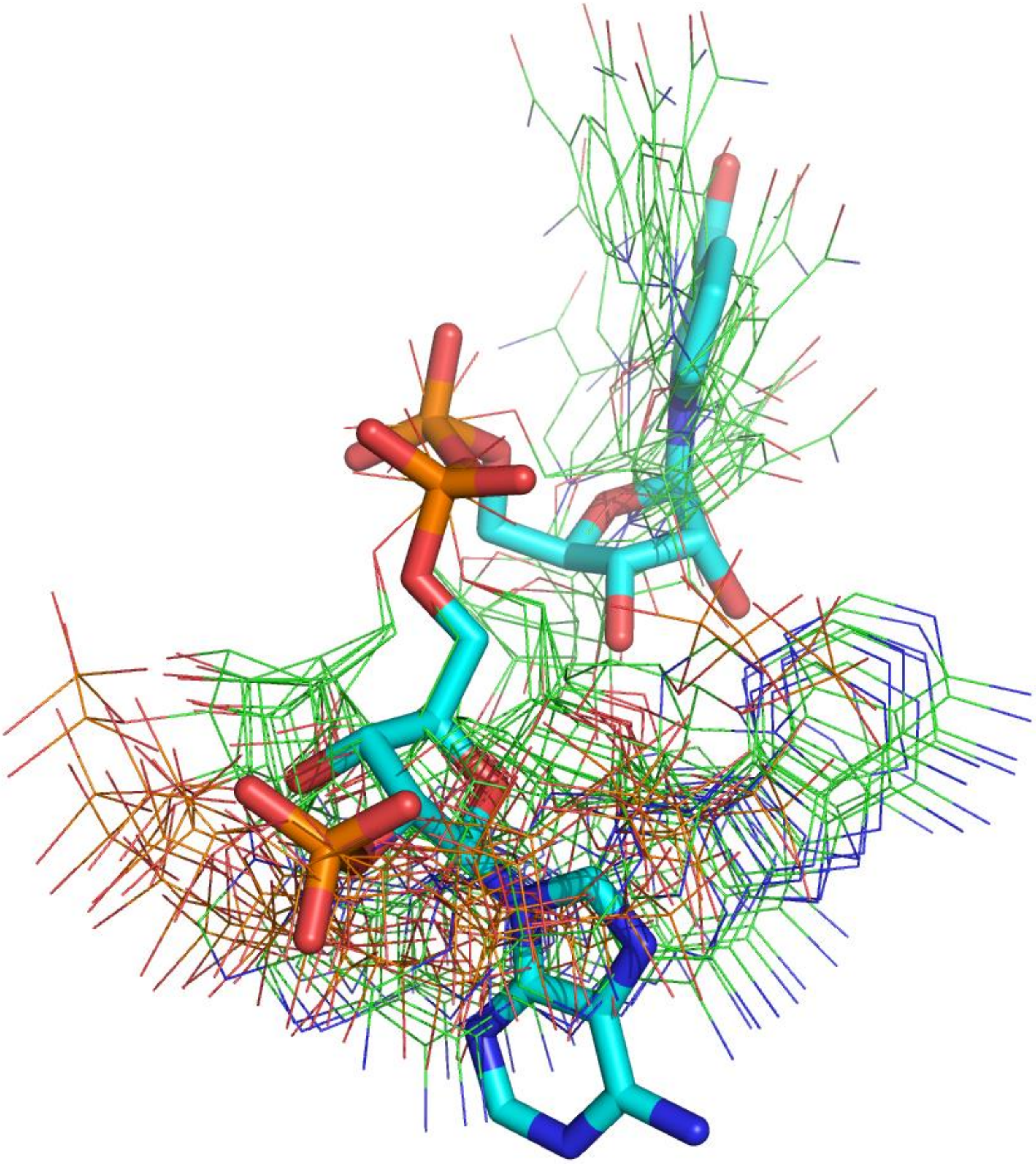

Supplementary Figure 8: Conformers of NADP molecule used for RosettaLigand Dock analysis. Molecule in ‘sticks’ (cyan) is borrowed from crystal structure 2JL1. Another 884 conformers (green lines) were generated by sampling 13 rotatable dihedrals ±30° from the dihedrals found in crystal structure.
